# Integrative multi-omics analysis reveals the drug-protein-ceRNA regulatory network in acute ischemic stroke

**DOI:** 10.3389/fmolb.2026.1779905

**Published:** 2026-04-09

**Authors:** Xiaoli Kong, Zhengyu Chen, Han Lu, Xueting Gao, Xiaoyu Zhou, Hao Su, Jun Teng, Haimeng You, Hongyan Ge

**Affiliations:** 1 Procurement and Supply Center, Yancheng TCM Hospital Affiliated to Nanjing University of Chinese Medicine, Yancheng, China; 2 Department of Neurology, Yancheng TCM Hospital Affiliated to Nanjing University of Chinese Medicine, Yancheng, China; 3 Department of Clinical Laboratory, The Affiliated Suzhou Hospital of Nanjing Medical University, Suzhou Municipal Hospital, Gusu School, Nanjing Medical University, Suzhou, China; 4 Department of Clinical Laboratory, Yancheng TCM Hospital Affiliated to Nanjing University of Chinese Medicine, Yancheng, China; 5 Department of Radiology, Yancheng TCM Hospital Affiliated to Nanjing University of Chinese Medicine, Yancheng, China

**Keywords:** acute ischemic stroke, ceRNA network, molecular docking, non-coding RNA, transcriptome

## Abstract

Previous studies have shown that non coding RNA (ncRNA) is closely related to the occurrence and development of acute ischemic stroke (AIS), but its systemic regulatory disease profile has not been fully elucidated. We collected peripheral whole blood samples from AIS patients and healthy controls for transcriptome expression profiling analysis of mRNA, micro RNA, and long non coding RNA (lncRNA). In transcriptome data analysis, differentially expressed RNAs were identified, and key functional pathways and microenvironmental changes in AIS were comprehensively analyzed. Based on the ceRNA hypothesis, a competitive regulatory network of ceRNA (lncRNA/miRNA/mRNA) for AIS disease occurrence was constructed. In downstream analysis, the upregulated mRNA in the ceRNA network was combined with drug target molecular information in the Drugbank database to screen and identify three direct targets, NFKBIA, TNFAIP6, and ORM1, all of which play key immunomodulatory and anti-inflammatory roles in the pathological process of AIS; Further combining with the PPI network, FN1 and MMP9 were identified as key predictive targets. Constructing a multi-level and multi-omics network map of drug protein ceRNA to explore the transformation pathway from molecular mechanisms to clinical drug targets. Molecular docking simulation was used to verify that the predicted targets FN1 and MMP9 can bind to current therapeutic drugs such as Acetylsalicylic acid, suggesting the possibility of FN1 and MMP9 as new targets for AIS treatment. This study follows a systematic strategy from constructing transcriptome regulatory networks to downstream clinical drug target validation, providing a new perspective for the occurrence and development of AIS diseases from the RNA regulatory level. The multi-omics landscape reveals potential molecular mechanisms and lays a solid theoretical foundation for identifying novel and reliable diagnostic biomarkers and potential therapeutic targets.

## 1 Introduction

Ischemic stroke (IS) is a general term for local cerebral blood circulation obstruction that causes neurological dysfunction (Jauch et al., 2013). Acute ischemic stroke (AIS) accounts for over 70% of IS cases, and its core pathological feature is sudden interruption of cerebral blood flow, leading to local cerebral tissue ischemia and hypoxic necrosis. It is characterized by high incidence, disability, and mortality rates. Despite advances in imaging and acute management, the therapeutic window for AIS is narrow, and current interventions offer limited benefit for long-term prognosis. Given the severe burden of AIS, thoroughly elucidating the molecular mechanisms underlying its onset, progression, and treatment has become a primary research imperative.

Recent advances in non-coding RNA (ncRNA) research have provided new directions for elucidating disease mechanisms. ncRNAs, which comprise the majority of the human transcriptome, mainly include microRNAs (miRNAs), long non coding RNAs (lncRNAs), and circular RNAs (circRNAs). They function as critical mediators of post-transcriptional regulation by modulating mRNA stability and translation efficiency. Furthermore, they govern critical pathological processes in stroke, including apoptosis, inflammation, and neuroprotection (Wang et al., 2018; Li et al., 2024; Yin et al., 2014). Given that peripheral blood serves as an ideal source for biomarker discovery, numerous studies have reported the differential expression of ncRNAs in the peripheral blood of AIS patients, suggesting their potential as feasible diagnostic or prognostic biomarkers (Toor et al., 2025; Zuo et al., 2019; Zhang and Zhang, 2024).

However, current research on ncRNA in AIS still has significant limitations. First, most studies have only focused on single or a few ncRNA molecules, failing to reveal their synergistic regulatory mechanisms at the transcriptome level (Mu et al., 2024; Jiang et al., 2025; Li et al., 2021; Lyu et al., 2015). Second, there is a lack of deep integration and verification with clinical therapeutic targets or drug mechanisms, making it difficult to form a comprehensive mechanistic understanding from gene regulatory networks to disease onset, progression, and potential treatment (Kong et al., 2024; Chen and Wu, 2021; Cui et al., 2022). In this context, the proposal of the competing endogenous RNA (ceRNA) regulatory mechanism provides a new perspective for the systematic analysis of gene regulatory networks (Salmena et al., 2011). This hypothesis suggests that lncRNA and circRNA act as sponges that competitively share miRNA response elements (MREs) to bind miRNAs, thereby regulating the inhibitory effects of miRNAs on their target genes (Zhang and Zhang, 2024; Xu M. et al., 2025; Chen et al., 2023). By constructing a ceRNA network, it is possible to systematically reveal the associations and complex regulatory mechanisms involving multiple types of RNA molecules.

Therefore, in this study, we employed a systematic multi-omics strategy to build a bridge between RNA regulatory networks and clinical translation. First, by integrating clinical sample transcriptome data, we identified aberrantly expressed coding and non-coding RNAs, aiming to reveal key functional pathways and micro-environmental changes following AIS. Based on the ceRNA hypothesis, a competitive regulatory network (lncRNA/miRNA/mRNA) was constructed. Furthermore, to explore the translational potential from mechanisms to therapeutics, we integrated the ceRNA network with drug-target information from DrugBank and PPI networks to identify key predictive targets, which were subsequently validated by molecular docking simulations. Finally, a multi-level and multi-omics network landscape of drug-protein-ceRNA was constructed. In summary, this study provides a new perspective on the pathogenesis of AIS from the viewpoint of RNA regulation, laying a solid theoretical foundation for identifying novel diagnostic biomarkers and treatment strategies.

## 2 Materials and methods

### 2.1 Clinical specimens

This study adopts a case-control design. Thirteen patients with AIS admitted to Yancheng Traditional Chinese Medicine Hospital from December 2024 to March 2025, as well as seven healthy controls matched for age, gender, and major vascular risk factors, were included. The inclusion criteria for the case group are: acute onset with symptoms lasting ≤24 h, blood collection within 10 h of admission, combined with medical history, physical signs, and CT/MRI/MRA/CTA examination, meeting the diagnostic criteria of *the 2018 Chinese Guidelines for the Diagnosis and Treatment of Acute Ischemic Stroke*, age 25–90 years, and informed consent signed by the patient or guardian. Exclusion criteria include recent use of antithrombotic/lipid-lowering drugs, cerebral hemorrhage/tumors, severe hepatic or renal dysfunction, autoimmune diseases, malignant tumors, and inability to cooperate. All study subjects collected 8–10 mL of peripheral venous whole blood (EDTA-K2 anticoagulant), mixed well after collection, and immediately stored at −80 °C to avoid repeated freezing and thawing. This study was approved by the Medical Ethics Committee of Yancheng Traditional Chinese Medicine Hospital (Approval Number: KY231202).

### 2.2 RNA extraction, library construction, and data pre-processing

In this study, total RNA was extracted using TRNzol Universal Reagent. Small RNA sequencing was performed using NovaSeq 6,000, while lncRNA sequencing was performed using NovaSeq x plus. After confirming the integrity of RNA through quality inspection, Shenzhen Weike Meng Technology Group Co Ltd. was commissioned to perform whole transcriptome sequencing on whole blood samples. The LncRNA library was constructed using rRNA deletion method. After passing the library inspection, different libraries were pooled according to the effective concentration and target data volume requirements for dual end sequencing. The raw data was subjected to Fast QC quality control (Q30 ≥ 95%). The data quality was evaluated through dimensionality reduction, with mRNA and lncRNA analyzed using principal component analysis (PCA) and miRNA visualized using t-distributed Stochastic Neighbor Embedding (t-SNE). For the independent external cohort (GSE16561), data quality and intra-group homogeneity were assessed by intra-group Pearson correlation analysis after ComBat batch-effect correction.

### 2.3 Differential expression analysis

Differential expression analysis was performed using the edgeR package (v4.4.2) in R (Robinson et al., 2010) to process the raw count data of mRNA, miRNA, and lncRNA separately. Low-expression genes were filtered out, followed by data normalization and statistical model fitting. Differentially expressed mRNAs (DEmRNAs), miRNAs (DEmiRNAs), and lncRNAs (DElncRNAs) were identified based on the significance criteria of *P-value* < 0.05 and |log2 FC| > 1.

Heatmaps were generated using the pheatmap package (v1.0.12) (Kolde, 2015) to illustrate the expression profiles of differentially expressed genes. Additionally, volcano plots were constructed using the ggplot2 package (v3.5.1) (Wickham, 2009) to depict the statistical significance.

### 2.4 Functional and pathway enrichment analysis

Targeting relationships among miRNAs, mRNAs, and lncRNAs were predicted using the miRanda algorithm (Enright et al., 2003). Gene Ontology (GO) and KEGG pathway enrichment analyses were performed using the clusterProfiler package (Xu et al., 2024) on genes involved in the ceRNA regulatory axes (ceRNA1: lncRNA↑-miRNA↓-mRNA↑, and ceRNA2: lncRNA↓-miRNA↑-mRNA↓). The top 10 terms with the most significant statistical relevance were selected for visualization.

Gene Set Enrichment Analysis (GSEA) was performed on the DEmRNAs using the GSEABase package (Subramanian et al., 2005). Ridge plots were generated to visualize the density distribution of log2 FC values for core enriched genes within the most significant gene sets. For this analysis, annotated gene sets were retrieved from the MSigDB collections (including h.all-v2025.1, c2.cp.v2025.1, and c5.go.v2025.1). The significance threshold was set at a relaxed *P-value* < 0.25, with a maximum gene set size of 500. False Discovery Rate (FDR) correction was applied using the Benjamini–Hochberg (BH) method. Visualize enrichment curves by screening pathways closely related to stroke and statistically significant through keywords such as *hypoxia*, *inflammation*, *apoptosis*, etc.

Gene Set Variation Analysis (GSVA) was performed on the transcriptome expression matrix using the GSVA R package (Hnzelmann et al., 2013). The top 50 differentially expressed pathways were visualized using heatmaps.

### 2.5 Construction of protein-protein interaction (PPI) network

PPI networks for the upregulated and downregulated DEmRNAs were constructed separately using the STRING database (https://string-db.org/) (Damian et al., 2024), with a minimum interaction score threshold of 0.4. The PPI networks were visualized using Cytoscape software (Shannon et al., 2003).

### 2.6 Construction of the ceRNA network

The mRNA-miRNA and miRNA-lncRNA interactions were integrated to construct the ceRNA network. Chord diagrams were generated using the circlize package (Gu et al., 2014) to visualize the ceRNA network. The nodes (lncRNAs, miRNAs, and mRNAs) were arranged radially and the interactions were depicted as links connecting the corresponding RNAs. Distinct colors were assigned to differentiate RNA types. Finally, Gene Ontology Biological Process (GO-BP) and KEGG pathway enrichment analyses were performed on the mRNAs associated in the ceRNA network.

### 2.7 Feature gene screening and ROC validation

The ceRNA1 (lncRNA↑-miRNA↓-mRNA↑) sub-network was selected for in-depth investigation. To explore potential therapeutic implications, relevant drug target genes were identified from the DrugBank database (https://go.drugbank.com/indications/DBCOND0030340#targets) (Wishart et al., 2008). Target proteins were annotated using the UniProt and PDB databases to establish the corresponding protein-gene mappings. Subsequently, the mRNAs within the ceRNA1 network were intersected with the identified AIS drug targets and visualized using Venn diagrams (Supplementary Tables S40, S41).

Furthermore, a PPI network was integrated to screen for feature genes associated with the intersecting targets, serving as candidate targets for predicting novel gene-protein-drug therapeutic axes. The interaction network was visualized and refined using the online platform ChiPlot (Steichen, 2001).

Finally, the diagnostic performance of predicted genes was validated using the pROC package (v1.18.5) (Robin et al., 2011). To ensure the reliability of the inferences, two independent public cohorts (GSE16561 and GSE58294) were introduced for external validation. A 5-gene diagnostic panel was constructed using logistic regression, and the Area Under the Curve (AUC) values and 95% confidence intervals (CI) were calculated to assess the reliability of the diagnostic models.

### 2.8 Construction of multi-omics network landscape

A Sankey diagram of the Drug-Protein-ceRNA regulatory network was constructed using the ggalluvial package (Brunson, 2017). In this diagram, streams represent regulatory interactions, illustrating the regulatory flow from therapeutic drugs to the ceRNA network.

### 2.9 Molecular docking simulation

Molecular docking simulations were performed to identify the interactions between four widely used therapeutic drugs for AIS/IS (Acetylsalicylic acid, Dipyridamole, Losartan, and Nicardipine) and the predicted targets. For the ligands, the 3D conformer SDF files of the drug structures were retrieved from PubChem (Sunghwan et al., 2016). For the receptors, the crystal structures of the target proteins were obtained from the RCSB PDB database (Burley et al., 2022), with selection criteria including X-ray diffraction, a resolution of <2.5 Å, and prioritizing crystal structures. The small molecules were processed using obgui (v3.1.1) (O'Boyle et al., 2011) for format conversion, 3D conformation generation, hydrogenation, and charge assignment to obtain standardized molecular structures. Protein pre-treatment, including the removal of water molecules and impurities as well as hydrogenation, was performed using PyMOL (v3.1.3) (Delano WLJPSF and Bioinformatics, 2002).

Subsequently, molecular docking simulations were conducted using AutoDock Vina (v1.5.7) (Trott and Olson, 2009) to predict the binding affinity between the drug molecules and the target proteins. The docking results were visualized using PyMOL.

### 2.10 Statistical analyses

All statistical analyses were performed using the R software environment (v4.2.0). The data visualization was conducted using the ggplot2 package in R and Cytoscape software. The STRING database was used to construct the PPI networks. Unless otherwise specified, a *P-value* < 0.05 was considered statistically significant. Due to the relatively small sample size of the exploratory cohort, a nominal P < 0.05 threshold was used to identify a comprehensive set of potential diagnostic markers. To stringently confirm the robustness of our core findings, a stricter threshold of False Discovery Rate (FDR) < 0.05 was subsequently applied to evaluate the key hub genes.

## 3 Results

### 3.1 Data quality assessment and differential expression analysis

Differentially expressed genes (DEGs) facilitate the discovery of critical disease-related genes, offering valuable insights for early diagnosis, the identification of therapeutic targets, and the formulation of personalized treatment regimens (Rosati et al., 2024).

**FIGURE 1:**
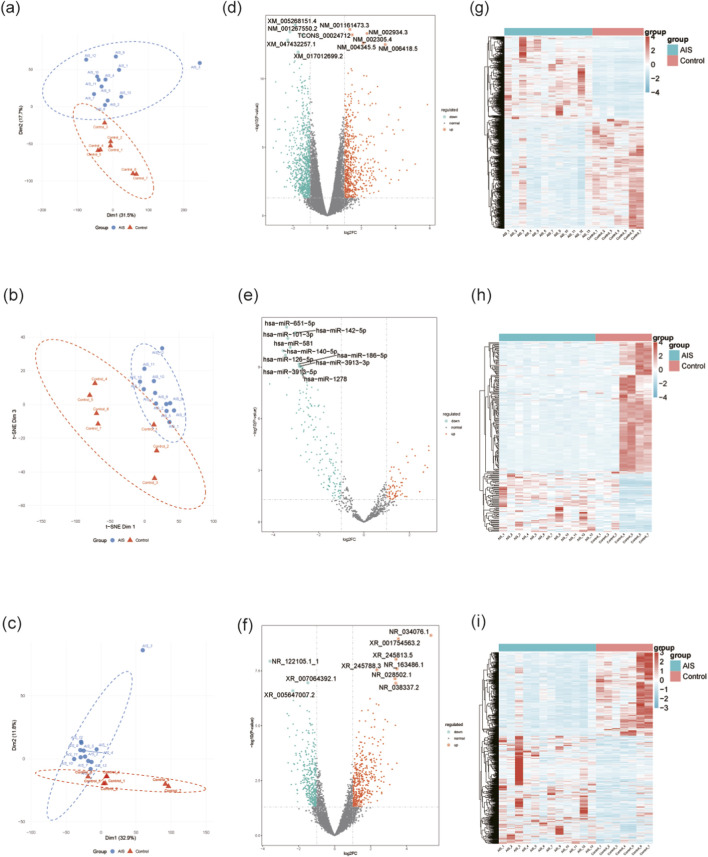
Whole-transcriptome differential expression analysis. **(a–c)** Sample quality control and dimensionality reduction. **(a)** PCA plot of mRNA; **(b)** t-SNE plot of miRNA; **(c)** PCA plot of lncRNA. **(d–f)** Volcano plots of differentially expressed mRNAs, miRNAs, and lncRNAs. The horizontal dashed line represents the significance threshold of P-value < 0.05. The vertical dashed lines indicate the fold-change threshold of |log2FC| > 1. Upregulated and downregulated RNAs are highlighted in red and green, respectively. **(g–i)** Heatmaps of the differentially expressed mRNAs, miRNAs, and lncRNAs. The color scale indicates normalized expression levels (red: high; green: low).

To evaluate data reliability and sample homogeneity, we conducted dimensionality reduction analysis on the transcriptome datasets. As shown in Figures 1a–c, the AIS and control groups were clearly separated in the reduced dimensional space, indicating significant differences in transcriptome features between the two groups and verifying the reliability of the data. Except for AIS_8, which is a significant outlier, the remaining samples demonstrated strong intra-group clustering, reflecting a reasonable experimental design and high biological reproducibility. To ensure the accuracy of subsequent analysis, the outlier sample AIS_8 was excluded. Furthermore, strong intra-group correlations were confirmed in the batch-corrected GSE16561 cohort (AIS r > 0.88; Control r > 0.92, Supplementary Figures S1, S2).

Differential expression analysis identified a total of 1,406 differentially expressed mRNAs (DEmRNAs; 605 upregulated, 801 downregulated), 231 differentially expressed miRNAs (DEmiRNAs; 71 upregulated, 160 downregulated), and 759 differentially expressed lncRNAs (DElncRNAs; 428 upregulated, 331 downregulated) in the AIS group compared to healthy controls. Volcano plots were generated to visualize the DERNAs (Figures 1d–f). Furthermore, heatmaps illustrated the distinct expression profiles of DERNAs between the AIS and control groups (Figures 1g–i). Notably, miRNAs exhibit a significant downregulation bias, with a much higher number of downregulated miRNAs than upregulated ones. We speculate that these downregulated miRNAs may play a more critical regulatory role in the pathogenesis of stroke.

### 3.2 Functional and pathway enrichment analysis

We further constructed an lncRNA/miRNA/mRNA network and performed GO and KEGG enrichment analyses to elucidate the regulatory mechanisms of candidate RNAs in AIS. Given that miRNAs have been shown to negatively regulate gene expression by binding to target mRNAs to induce degradation or inhibiting translation, the regulatory network was constructed using opposite expression patterns (Li et al., 2025).

**FIGURE 2:**
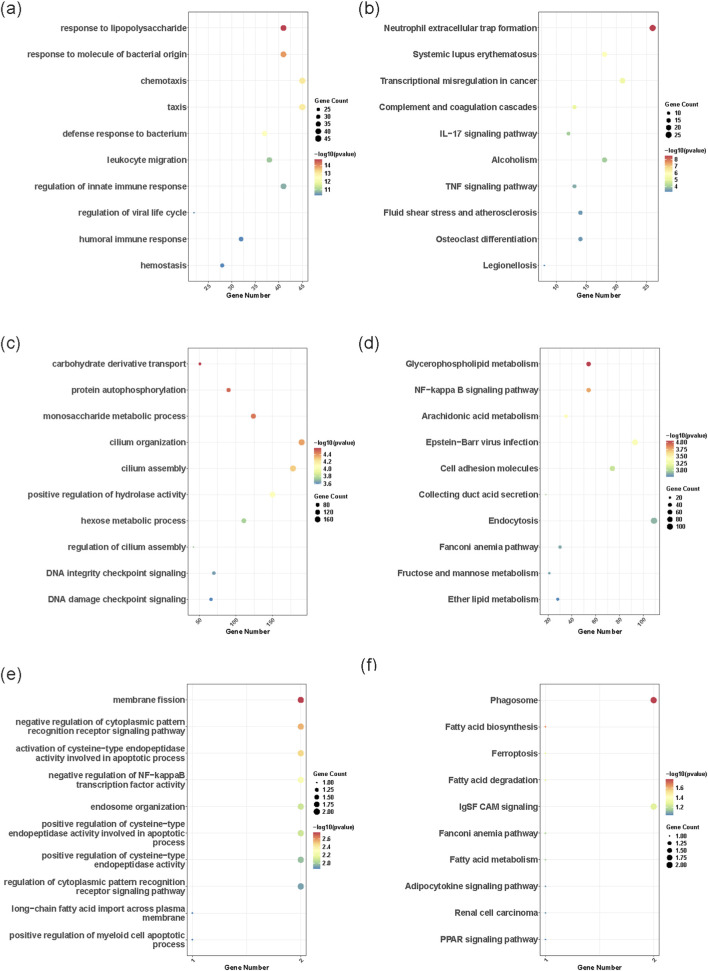
**(a)** upmRNA_GO: Biological process. **(b)** upmRNA_KEGG pathway. **(c)** downmiRNA_GO: Biological process. **(d)** downmiRNA_KEGG pathway. **(e)** upIncRNA_GO: Biological process. **(f)** upIncRNA_KEGG patheay.

For the upregulated mRNAs, Gene Ontology Biological Process (GO-BP) analysis (Figures 2a,b) showed that upregulated genes were mainly enriched in immune and inflammatory responses, with a focus on neutrophil-mediated processes (e.g., response to lipopolysaccharide and chemotaxis). In the KEGG pathway analysis, Neutrophil Extracellular Trap formation (NETosis) was the most significantly enriched pathway (*P_adjust* < 0.003). Additionally, the Complement and coagulation cascades and the IL-17 signaling pathway were also implicated. These results suggest that ischemic injury in AIS induces strong immune activation, and NETosis may serve as a critical mechanism for secondary brain injury (Oh et al., 2025; Gao et al., 2024; Li C. et al., 2022).

For the downregulated miRNAs, functional enrichment analysis of their target genes (Figures 2c,d) suggested that downregulated miRNAs may affect core pathological processes by releasing target gene inhibition. GO-BP analysis showed primary enrichment in cellular energy metabolism (e.g., carbohydrate derivative transport) and cellular communication regulation (e.g., protein autophosphorylation). The KEGG pathway analysis highlighted enrichment in glycerophospholipid metabolism, the NF-κB signaling pathway, and arachidonic acid metabolism. The results suggest that the downregulated miRNAs may exacerbate post-stroke metabolic dysregulation and amplify inflammatory signaling (e.g., NF-κB) by relieving the inhibition of these critical pathways (Xu M. et al., 2025; González-López et al., 2023; Kanki et al., 2023).

For the upregulated lncRNA, GO-BP analysis of their target genes (Figures 2e,f) revealed that they were mainly enriched in cell apoptosis and immune negative feedback. GO-BP enrichment was observed in terms such as negative regulation of cytoplasmic pattern recognition receptor signaling pathway and negative regulation of NF−kappaB transcription factor activity. The KEGG analysis showed enrichment in the Phagosome, IgSF CAM signaling, indicating that upregulated lncRNAs may play a complex regulatory role in cerebral ischemic injury by finely regulating the balance between apoptosis and necroptosis, inhibiting the overactivation of intrinsic immune signals such as NF-κB (Gao et al., 2021; Xu et al., 2016; Yang et al., 2022).

In summary, although these differentially expressed molecules belong to different RNA types, they exhibit a high degree of functional synergy. The upregulated mRNAs directly reflect immune activation. The downregulated miRNAs further release metabolic and inflammatory signals via a derepression mechanism. The upregulated lncRNA is implicated in the regulation of cell death modes. This inherent consistency in functionality not only reveals the core pathological mechanism of AIS, but also strongly supports the rationality of constructing a ceRNA network in our subsequent analysis.

### 3.3 Gene set enrichment analysis (GSEA) and gene set variation analysis (GSVA)

To systematically evaluate the dynamic changes in gene expression profiles after AIS and identify key biological processes and signaling pathways, we conducted gene set enrichment analysis (GSEA) and gene set variation analysis (GSVA) on DEmRNAs.

**FIGURE 3:**
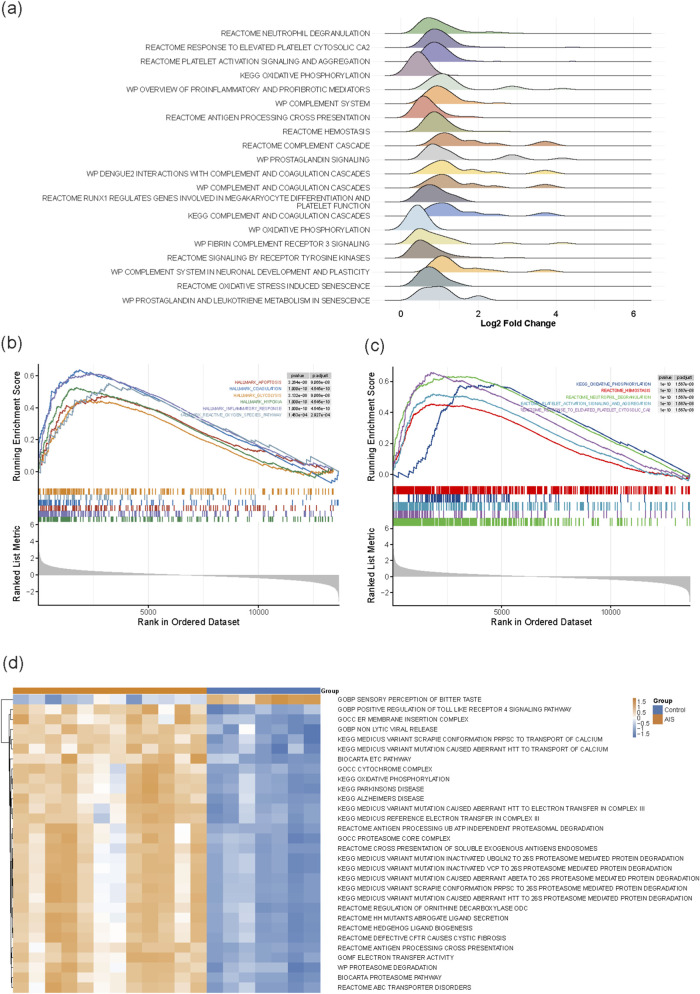
**(a)** Pathway enrichment density. **(b)** Major pathological states in AIS. **(c)** Activation of molecular cascades. **(d)** Top 30 signigicent pathways in AIS vs Control.

The density distributions visualized by the ridge plot (Figure 3a) revealed a significant rightward shift (Log2FC > 0) for pathways such as neutrophil degranulation, platelet activation signaling and aggregation. This indicates that the core molecules within these pathways are generally upregulated in the AIS group. Furthermore, pathways related to oxidative phosphorylation and complement and coagulation cascades exhibited consistent positive shifts. These results suggest a coordinated enhancement of immune-related processes and platelet activation following AIS, accompanied by distinct alterations in metabolic pathways.

Hallmark gene set analysis (Figure 3b) revealed that pathology-related pathways, including Apoptosis, Hypoxia and Inflammatory Response were significantly upregulated in the blood transcriptomes of AIS patients (FDR <0.05). Canonical Pathways (C2:CP) database (Figure 3c) highlighted the significant activation of platelet activation signaling and aggregation, neutrophil degranulation, and oxidative phosphorylation. The above results are highly consistent with the characteristics of inflammation, coagulation activation, and cellular injury identified in the DEmRNA functional enrichment analysis, confirming the core pivotal driving role of immuno inflammatory and coagulation responses in the pathogenesis of AIS at the pathway level.

The heatmap showed that, consistent with the GSEA results, mitochondrial energy metabolism pathways (e.g., KEGG-OXIDATIVE-PHOSPHORYLATION) exhibited high activity (upregulation) in the AIS group, which may reflect the high metabolic demands of peripheral immune cells after AIS. Furthermore, pathways related to neurodegenerative diseases, such as Parkinson’s disease and Alzheimer’s disease, were also enriched in the AIS group. This implies the existence of shared molecular mechanisms between AIS-induced neural injury and neurodegenerative pathologies.

### 3.4 Protein-protein interaction (PPI) and ceRNA network

**FIGURE 4:**
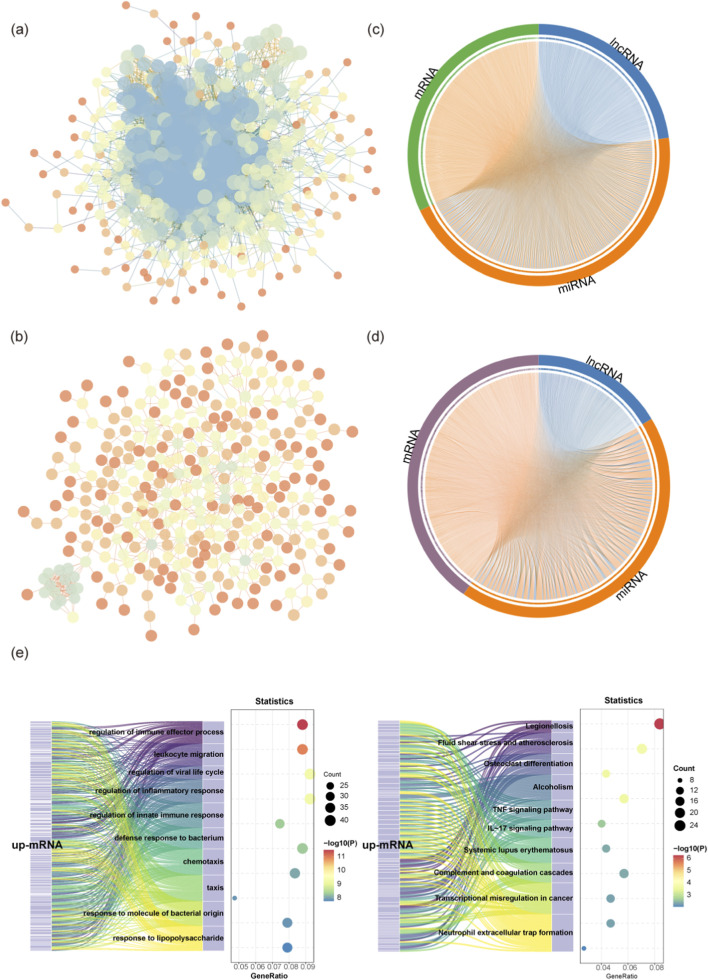
PPI networks and the ceRNA network analysis. **(a,b)** Protein-Protein Interaction (PPI) networks. The interaction networks of **(a)** upregulated DEmRNAs and **(b)** downregulated DEmRNAs. Nodes represent proteins, and edges represent potential interactions. **(c,d)** Chord diagrams of the ceRNA networks. **(c)** The ceRNA1 network (upregulated axis: up-mRNA/down-miRNA/up-lncRNA). **(d)** The ceRNA2 network (downregulated axis: down-mRNA/up-miRNA/down-lncRNA). **(e)** Functional enrichment analysis of the ceRNA1 network. Sankey diagrams displaying the GO Biological Process (left) and KEGG Pathway (right) enrichment results for the mRNAs involved in the ceRNA1 axis. The flows trace the linkage between specific genes (mRNAs) and the enriched functional terms.

The occurrence of AIS is not attributable to a single gene, but by the complex regulatory effects of multiple genes working together. Consequently, we constructed PPI networks of the identified DEmRNAs (Figures 4a,b), which revealed the abnormal expression protein interaction module of AIS pathogenesis.

Based on the ceRNA theory, we constructed two sub-networks with opposite regulatory patterns, including the ceRNA1 network (lncRNA↑-miRNA↓-mRNA↑) and the ceRNA2 network (lncRNA↓-miRNA↑-mRNA↓). The complex targeting relationships were visualized using chord diagrams (Figures 4c,d). Given that the ceRNA2 network failed to exhibit significant enrichment in AIS-specific pathological pathways, we selected the ceRNA1 network for in-depth investigation. Functional enrichment analysis of mRNA in the ceRNA1 network (Figure 4e) revealed that the GO-BP biological process was significantly enriched in immune related pathways, responding to lipopolysaccharides and defending against bacteria. The results suggest that ischemic injury involves the dynamic regulation of inflammation and the chemotactic recruitment of immune cells. KEGG pathway analysis showed that the TNF and IL-17 signaling pathways, as well as the complement and coagulation cascades, were highly enriched, while neutrophil extracellular traps (NETosis) were significantly enriched. These results suggest that the ceRNA1 axis may serve as a pivotal regulatory network driving post-ischemic inflammation and secondary tissue injury in AIS.

### 3.5 Identification of predicted targets, ROC validation and construction of the drug-protein-ceRNA axis

**FIGURE 5:**
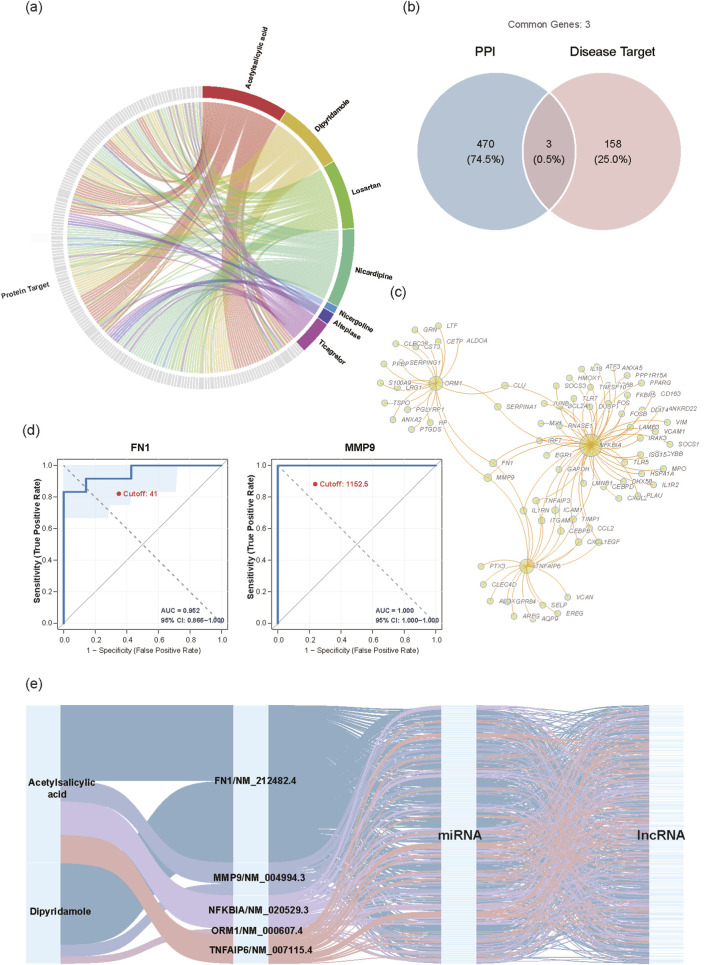
Identification of predicted targets, ROC Validation and construction of the drug-protein-ceRNA axis. **(a)** Drug-target interaction network in AIS. **(b)** Venn diagram of the intersection between PPI hub genes and disease-related targets. **(c)** PPI sub-network of the key feature genes. Nodes represent proteins, and edges denote interactions; hub genes are highlighted. **(d)** ROC curves evaluating the diagnostic performance of the two candidate genes (FN1 and MMP9) in AIS. The Area Under the Curve (AUC) indicates diagnostic accuracy. **(e)** Sankey diagram of the Drug–Protein–mRNA–miRNA–lncRNA regulatory axis. The flows illustrate the potential molecular mechanisms by which candidate drugs target the ceRNA network.

To explore the clinical translational potential of the ceRNA1 network and construct a comprehensive therapeutic landscape, we performed an intersection analysis between the identified upregulated DEmRNAs and known AIS drug targets from the DrugBank database (Figures 5a,b). Figure 5a provides a visualization of known drug-target interactions in AIS, while the Venn diagram identified three direct drug targets: NFKBIA, TNFAIP6, and ORM1. Given the functional synergy of proteins, we further constructed a PPI sub-network centered around the 3 direct targets (Figure 5c). We found that both FN1 and MMP9 have close physical interactions with the direct targets, suggesting a critical synergistic regulatory role in drug response pathways. Therefore, they were included as predictive targets in the study. ROC curve analysis validated the clinical potential of these feature genes (Figure 5d), with MMP9 (AUC = 1.000) and FN1 (AUC = 0.952), exhibiting extremely high sensitivity and specificity. To mitigate small-sample bias, we validated these signatures in two independent cohorts (GSE16561, GSE58294). While MMP9, ORM1, and TNFAIP6 maintained robust diagnostic performance, FN1 and NFKBIA showed variable performance across cohorts. Notably, the 5-gene panel achieved outstanding AUCs of 0.853 and 0.943 in GSE16561 and GSE58294, respectively (Supplementary Figure S3).

Based on the above findings, we integrated drugs, core targets (direct and predictive), and downstream ncRNAs to construct a multi-omics regulation Sankey diagram of drug protein/mRNA-miRNA-lncRNA (Figure 5e). The visualization revealed the hierarchical molecular mechanisms by which drugs (primarily Acetylsalicylic acid and Dipyridamole) intervene in the pathogenesis of AIS by targeting key gene clusters and subsequently regulating the downstream miRNA-lncRNA axes.

### 3.6 Molecular docking simulation

**FIGURE 6:**
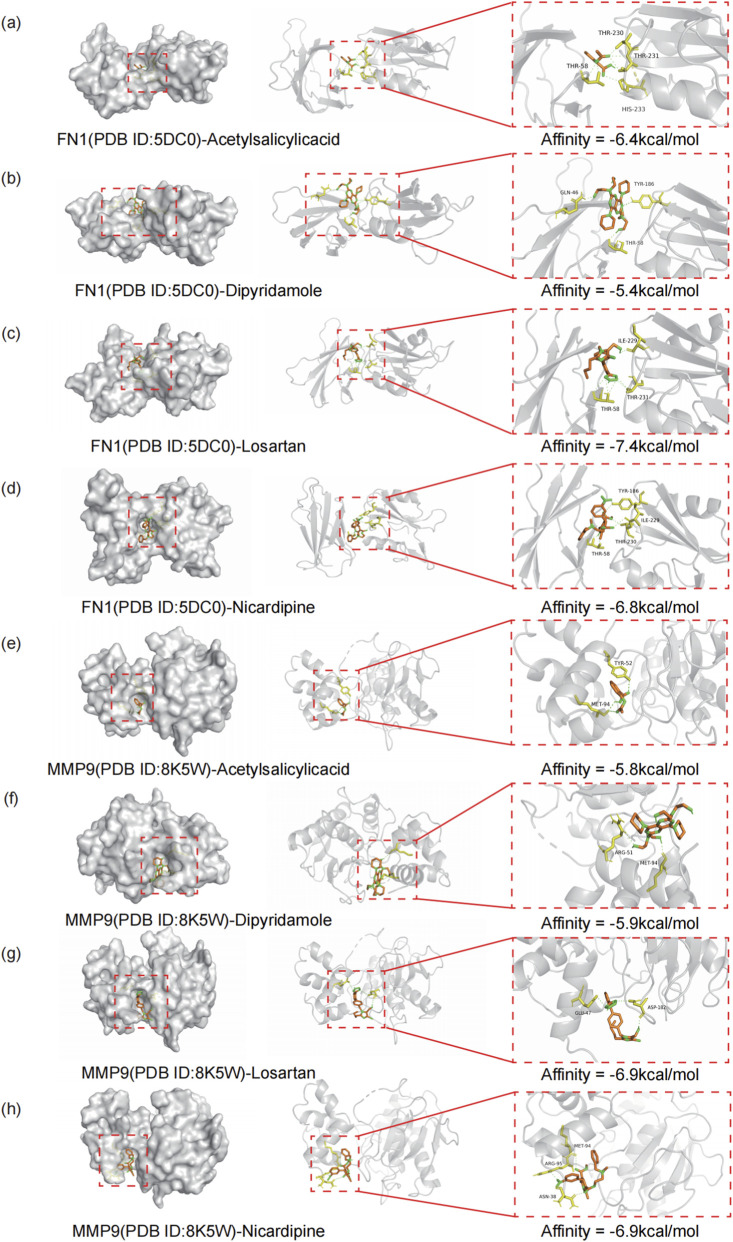
Molecular docking simulation of predicted targets with therapeutic drugs. **(a–d)** Molecular docking complexes for FN1 (PDB ID: 5DC0) with four potential therapeutic drugs: **(a)** Acetylsalicylic acid, **(b)** Dipyridamole, **(c)** Losartan, and **(d)** Nicardipine. **(e–h)** Molecular docking complexes for MMP9 (PDB ID: 8K5W) with the same four potential therapeutic drugs: **(e)** Acetylsalicylic acid, **(f)** Dipyridamole, **(g)** Losartan, and **(h)** Nicardipine. The calculated binding affinity (kcal/mol) for each ligand-receptor complex is indicated in the corresponding panel. The images depict the predicted binding modes and key interacting residues.

To further validate the feasibility of the predicted targets (FN1 and MMP9) as drug binding sites from a structural biology perspective, we performed molecular docking simulations (Figure 6). Using a binding energy ≤ −5.0 kcal/mol as the threshold for the existence of stable complexes (Wang et al., 2025). The analysis results showed that both MMP9 and FN1 could form thermodynamically stable complex structures with the target drug molecules. Specifically, MMP9 exhibited a strong binding affinity with the drug molecules, achieving a minimum binding energy of −7.4 kcal/mol. The binding energies for FN1 were all stronger than −5.8 kcal/mol, indicating that the drug molecules can effectively fit into the active pockets of these target proteins. These findings not only validate the physical basis of the interaction between FN1/MMP9 and AIS therapeutic drugs at the atomic level but also further support the reliability of our strategy to mine potential therapeutic targets via PPI networks, providing a solid theoretical foundation for drug development and mechanistic research.

## 4 Discussion

In this study, we constructed a ceRNA regulatory network for the occurrence and development of AIS disease through whole transcriptome sequencing and multi-omics integration analysis. The system revealed the molecular characteristics of ischemic injury. Functional enrichment analyses, corroborated by GSVA and GSEA results, consistently revealed significant immune activation and metabolic alterations in AIS patients. Specifically, these alterations included the activation of the complement coagulation cascade, enhancement of innate immune responses (e.g., neutrophil degranulation), and upregulation of energy metabolism processes (e.g., oxidative phosphorylation). These findings suggest that post-stroke transcriptional dysregulation is accompanied by alterations in downstream metabolic processes, which collectively exacerbate the systemic inflammatory response.

The ceRNA hypothesis suggests that lncRNA can competitively bind to miRNA to release its inhibitory effect on target mRNA (Li et al., 2021; Bai et al., 2017; Chen et al., 2018; Chen et al., 2026; Li G. et al., 2022; Li et al., 2017). The core ceRNA1 sub-network identified in this study revealed that widespread downregulation of miRNAs serves as a key mechanism leading to overexpression of AIS pathological genes. Notably, multiple key miRNAs in the ceRNA network have been widely validated in AIS mechanism research. For instance, the miR-19 family and hsa-miR-21 are closely associated with inhibiting apoptosis and promoting inflammatory responses (Yan et al., 2017; Barrera-Vázquez et al., 2022; Eyileten et al., 2018; Dhiraj et al., 2013; Ge et al., 2019). The hsa-miR-126 regulates endothelial homeostasis, vascular remodeling, and blood-brain barrier (BBB) integrity (Zhuang et al., 2025). Furthermore, the enrichment of the hsa-miR-142 family and hsa-miR-223–5p is highly consistent with the changes in neutrophil and T-cell subsets observed in our study, and are closely related to the activation mechanism of the NETosis pathway (Du et al., 2019; Curcio et al., 2023; Zhan et al., 2023). Meanwhile, the low expression of hsa-miR-146b-5p relieves the inhibition of the NF-κB pathway, thereby exacerbating neuroinflammation (Gaudet et al., 2017). Similarly, miRNA families such as hsa-miR-29b-3p and hsa-miR-101–3p can directly target matrix metalloproteinases (e.g., MMP9) or ECM components (e.g., FN1), thereby regulating BBB disruption and vascular remodeling (Dhiraj et al., 2013; Centa et al., 2021). Importantly, our study links these miRNAs to the functional pathway via the ceRNA network. This suggests that the downregulation of miRNAs in AIS patients may trigger a cascade of downstream inflammatory responses and BBB disruption.

The downstream analysis of this study translated the ceRNA network into clinical target prediction, focusing on the expression of key genes in AIS and their diagnostic and prognostic significance. By integrating the PPI network with the DrugBank database, we identified five characteristic genes, NFKBIA, TNFAIP6, ORM1, FN1, and MMP9. Notably, all these core hub genes remained significantly differentially expressed even under stringent multiple testing correction (FDR <0.05, Supplementary Figure S4). They are not only highly diagnostic biomarkers in our initial cohort (AUC>0.95), but also potential therapeutic targets. Importantly, large independent cohorts validated their robust diagnostic potential, with the 5-gene panel achieving an AUC up to 0.943. Notably, FN1 and NFKBIA showed non-significant fluctuations in the GSE58294 cohort, which is consistent with the time-dependent transcriptional dynamics and heterogeneous sampling windows following acute ischemic stroke (Li et al., 2011; Stamova et al., 2015). Multiple studies have confirmed that the five genes are significantly upregulated in AIS patients. Specifically, as an inflammation-related gene, NFKBIA has been shown to exhibit significantly elevated mRNA levels in the peripheral blood of acute AIS patients (Vibo et al., 2024), suggesting its critical role in the early inflammatory response. TNFAIP6 (TSG-6), a known anti-inflammatory protein, likely represents a compensatory protective mechanism against ischemic injury (Qu et al., 2022). ORM1, an acute-phase protein, is consistently upregulated in both proteomics datasets (e.g., PXD062958) and relevant literature, reflecting the systemic response to inflammation and tissue injury (Street et al., 2012; Wan et al., 2019). Additionally, FN1 expression increases during post-stroke extracellular matrix remodeling and vascular repair, implicating it in both BBB injury and restoration (Huang et al., 2015; Khan et al., 2012). Finally, MMP9 expression is markedly elevated in the plasma and brain tissue of AIS patients and is closely correlated with infarct volume, stroke severity, and prognosis (Xu L. et al., 2025; Ramos-Fernández et al., 2011; Qin et al., 2022). These ROC results strongly support the diagnostic potential of the identified hub genes; however, future large-scale prospective clinical cohorts will be required to fully confirm their clinical applicability.

Furthermore, our multi-omics Sankey diagram and molecular docking results reveal that classic antiplatelet drugs, Acetylsalicylic acid and Dipyridamole, may exert pleiotropic therapeutic effects by targeting the predictive targets. The discovery provides a theoretical basis for drug repurposing, grounded in structural biology and transcriptomics, and suggest that interventions targeting the downstream effector of the ceRNA axis may represent a promising novel strategy for AIS treatment.

Although this study systematically explored the potential mechanisms of AIS through a multi-omics approach, several limitations remain. First, the results are primarily based on bioinformatics predictions and computational simulations. The lack of direct *in vitro* and *in vivo* experimental validation for the specific lncRNA/miRNA/mRNA axes and drug-target interactions remains a common challenge in ceRNA network research. Second, the present study was based on a relatively small peripheral blood sample size, which results in limited statistical power and a potential risk of false-positive inferences. Although this captures the systemic inflammatory state, it may not fully represent the actual pathological processes occurring within brain tissue. To partially alleviate this concern, we validated our key findings using independent external cohorts; nevertheless, future large-scale, multi-center studies are still needed to improve the statistical robustness and clinical translation of these hub biomarkers.

## 5 Conclusion

This study utilized a multi-omics integration strategy to construct a AIS transcriptional regulatory landscape of peripheral blood and revealed a molecular regulatory network centered around the ceRNA1 axis (lncRNA↑-miRNA↓-mRNA↑). This network was identified as a critical driver of in the pathogenesis of AIS. More importantly, we translated this mechanism discovery into clinical treatment strategies through drug target network extension and molecular docking validation. We identified FN1 and MMP9 as predictive targets and revealed potential new mechanisms for classical drugs to target these molecules. In conclusion, this study not only elucidates the complex post-transcriptional regulatory mechanism of AIS but also provides a solid theoretical foundation and data support for precision diagnostic biomarker screening and drug repurposing strategies based on ceRNA networks.

## Data Availability

The datasets presented in this study can be found in online repositories. The names of the repository/repositories and accession number(s) can be found below: https://ngdc.cncb.ac.cn/search/specific?db=hra&q=HRA015811, HRA015811.
