# Supplementary material for: Integrative multi-omics analysis reveals the drug-protein-ceRNA regulatory network in acute ischemic stroke
Source: Front Mol Biosci. 2026 Apr 9;13:1779905. doi: 10.3389/fmolb.2026.1779905 (PMC13102545; doi:10.3389/fmolb.2026.1779905)
Supplement: Supplementary file 1 [file Supplementaryfile1.docx]

Supplementary Figures

**Integrative Multi-omics Analysis Reveals the Drug-Protein-ceRNA Regulatory Network in Acute Ischemic Stroke**

**
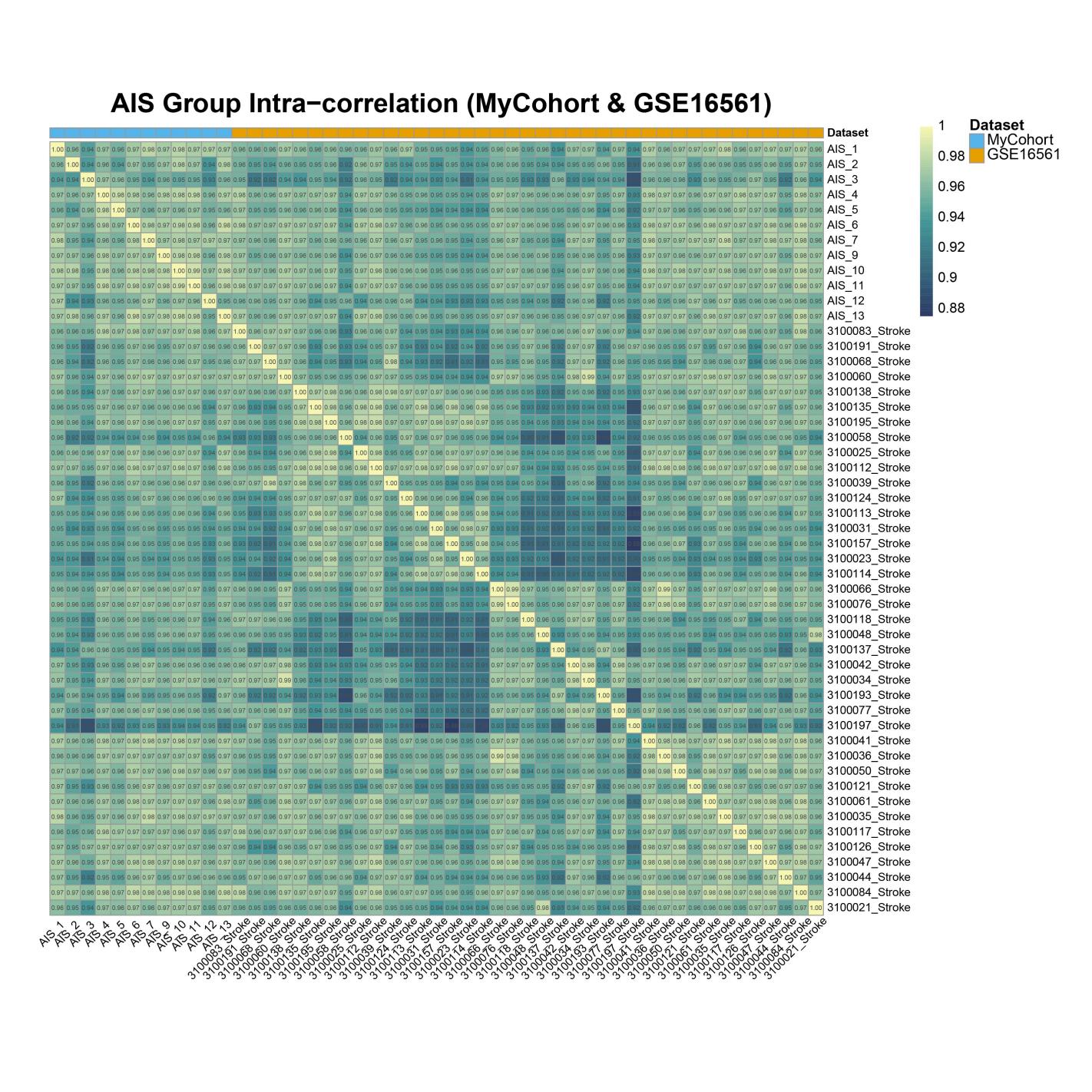
Supplementary Figure S1. Transcriptomic consistency of Acute Ischemic Stroke (AIS) samples between the local cohort and the independent validation cohort. The heatmap displays the intra-group Pearson correlation coefficients.**

**
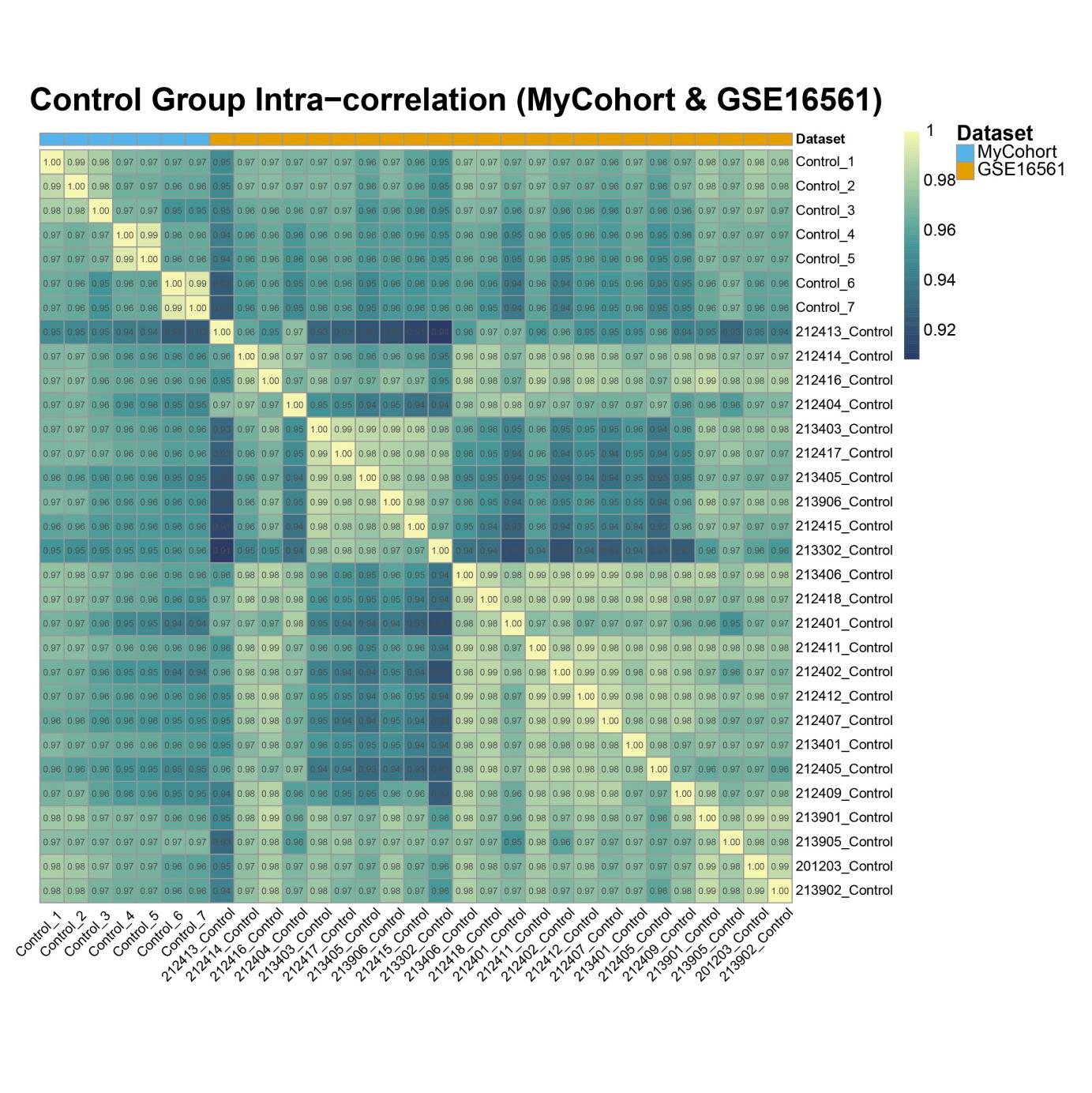
Supplementary Figure S2. Transcriptomic consistency of healthy Control samples between the local cohort and the independent validation cohort. The heatmap displays the intra-group Pearson correlation coefficients.**

**
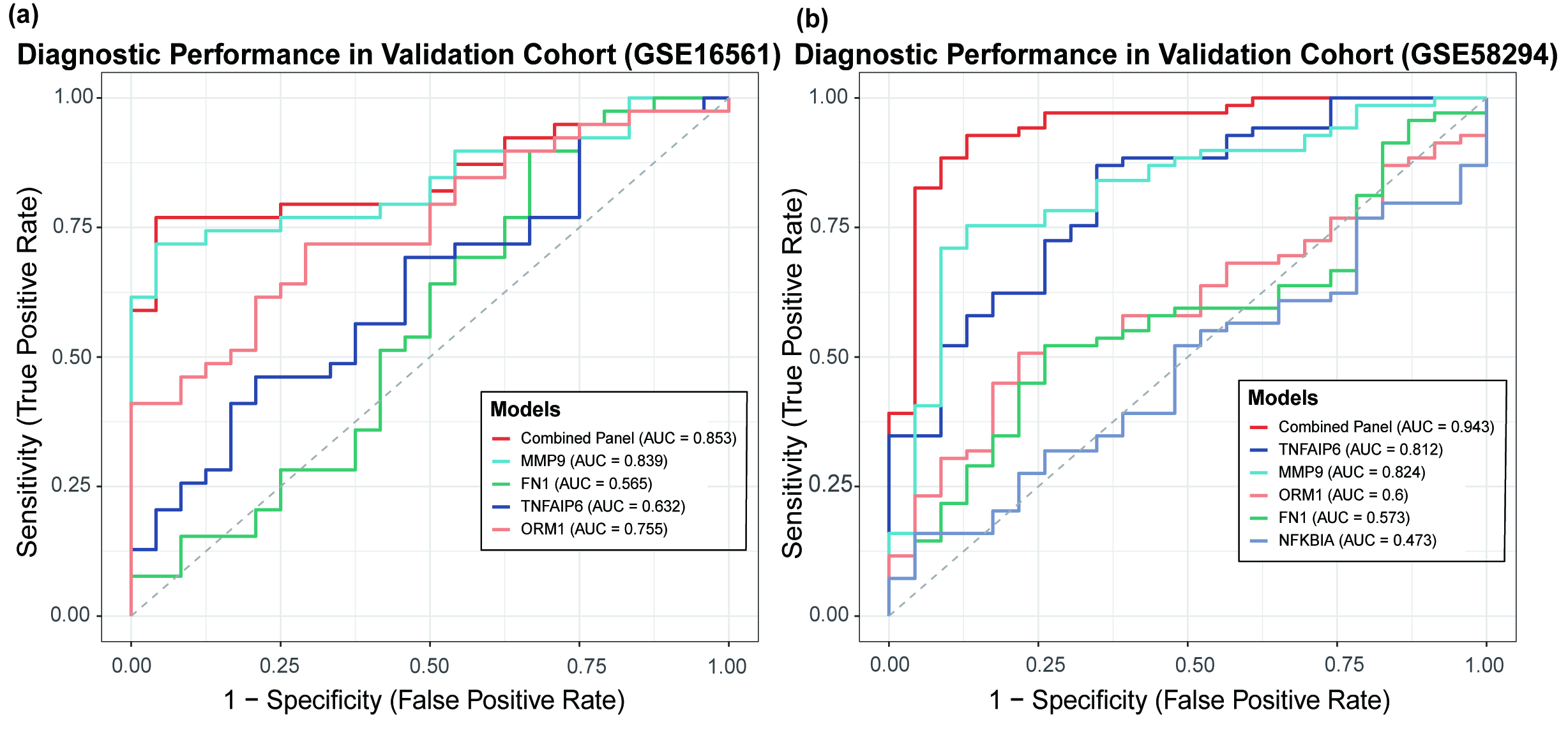
Supplementary Figure S3. Diagnostic performance of core hub genes and the 5-gene panel in independent validation cohorts. (a) Receiver operating characteristic (ROC) curves of the 5-gene panel and individual hub genes in the GSE16561 cohort. The panel achieved an AUC of 0.853; individual genes included MMP9 (AUC = 0.830), FN1 (AUC = 0.656), TNFAIP6 (AUC = 0.812), and ORM1 (AUC = 0.755). (b) ROC curves in the GSE58294 cohort. The 5-gene panel achieved an AUC of 0.943; evaluated genes included TNFAIP6 (AUC = 0.813), MMP9 (AUC = 0.804), ORM1 (AUC = 0.773), FN1 (AUC = 0.573), and NFKBIA (AUC = 0.473).**

**
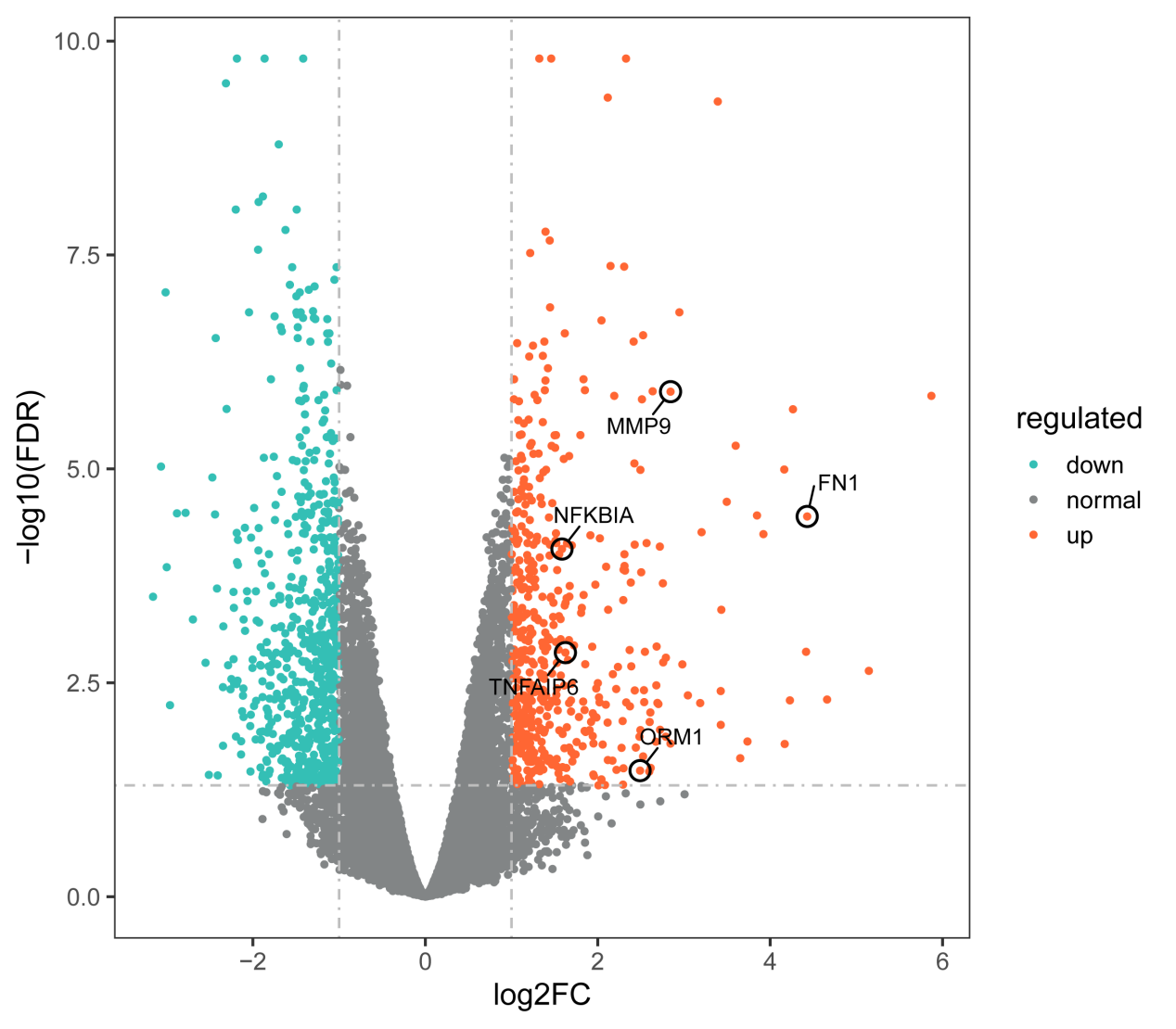
**

**Supplementary Figure S4. Volcano plot of differentially expressed genes based on False Discovery Rate (FDR) correction. The x-axis represents the log2(Fold Change), and the y-axis represents the -log10(FDR). Red and green points indicate significantly up-regulated and down-regulated genes, respectively (thresholds: FDR < 0.05 and |log2FC| > 1). Grey points represent non-significant genes. The five core hub genes (NFKBIA, TNFAIP6, ORM1, FN1, and MMP9) are explicitly highlighted with black open circles and text labels, demonstrating their robust statistical significance even after stringent multiple testing correction.**


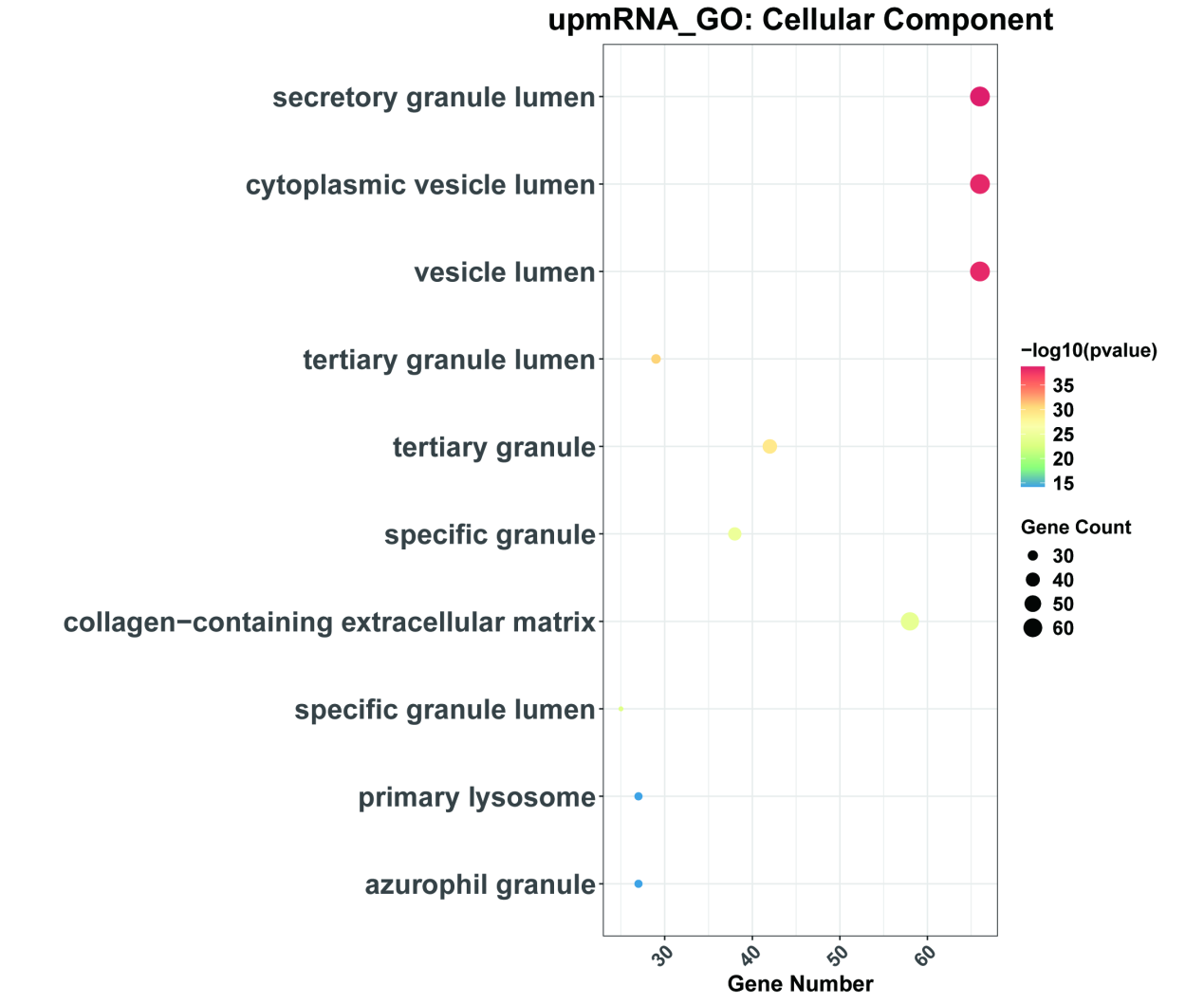
**Supplementary Figure S5. GO Cellular Component (CC) enrichment analyses for the upregulated mRNAs.**


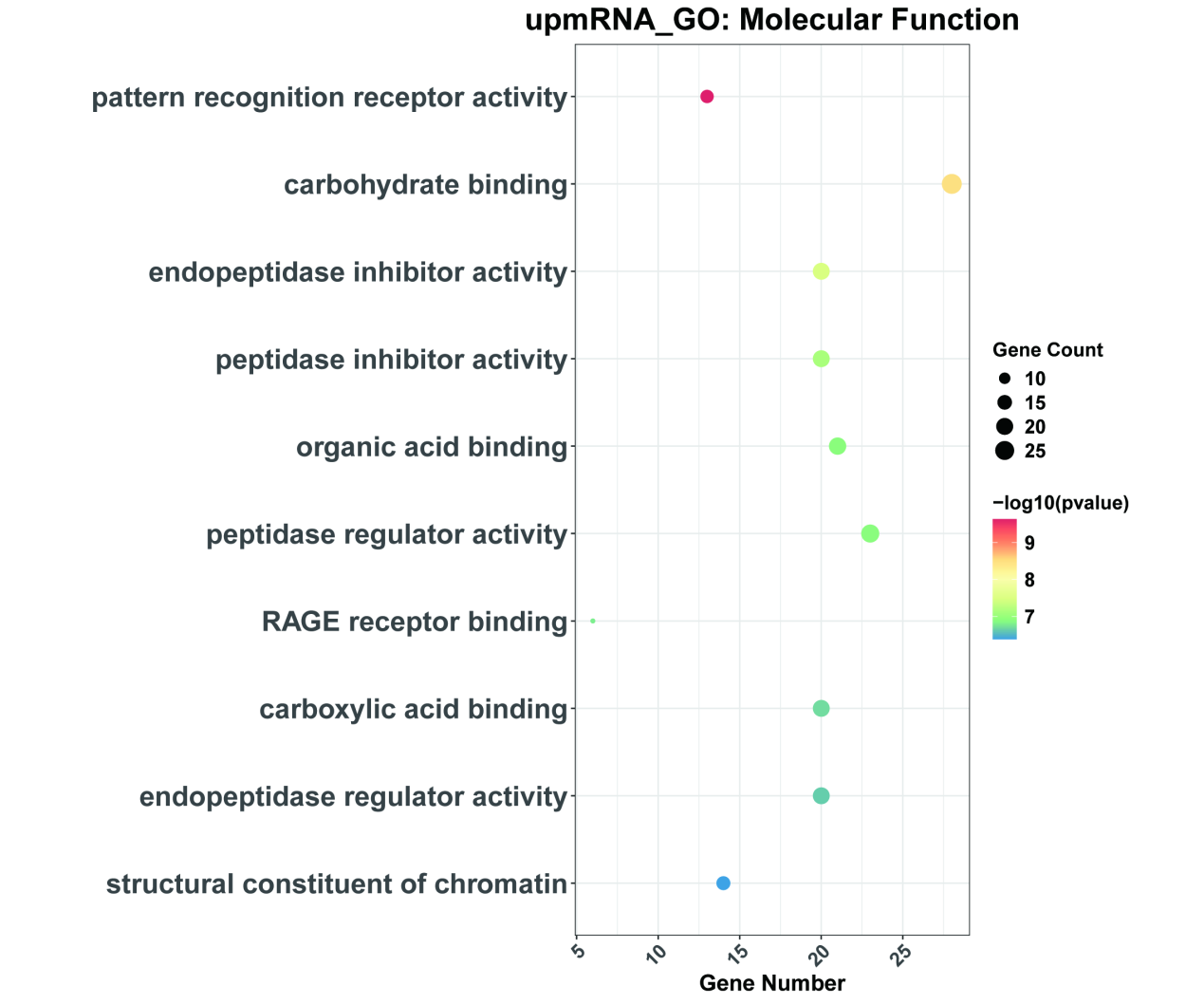
**Supplementary Figure S6. GO Molecular Function (MF) enrichment analyses for the upregulated mRNAs.**


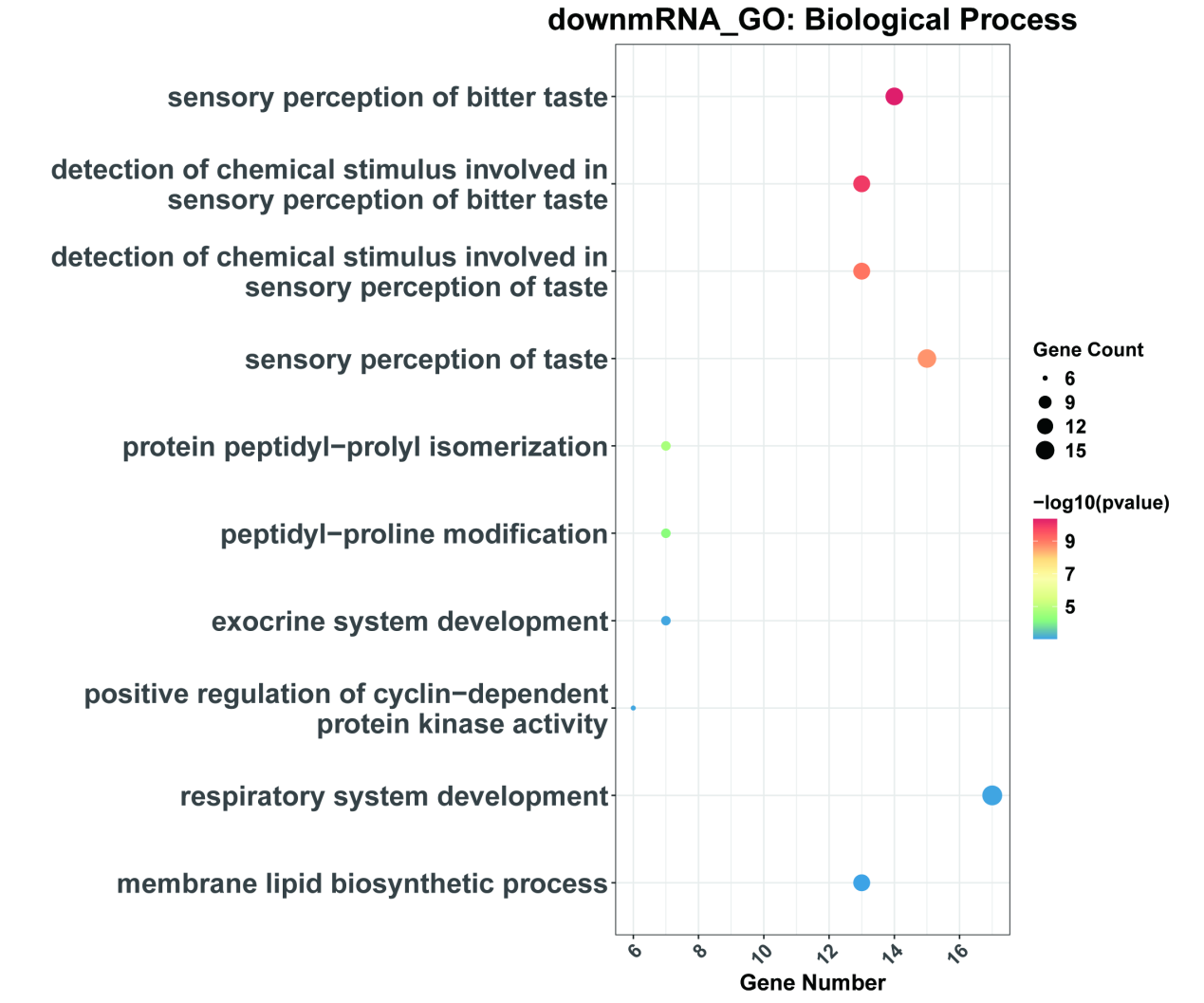
**Supplementary Figure S7. GO Biological Process (BP) enrichment analyses for the downregulated mRNAs.**


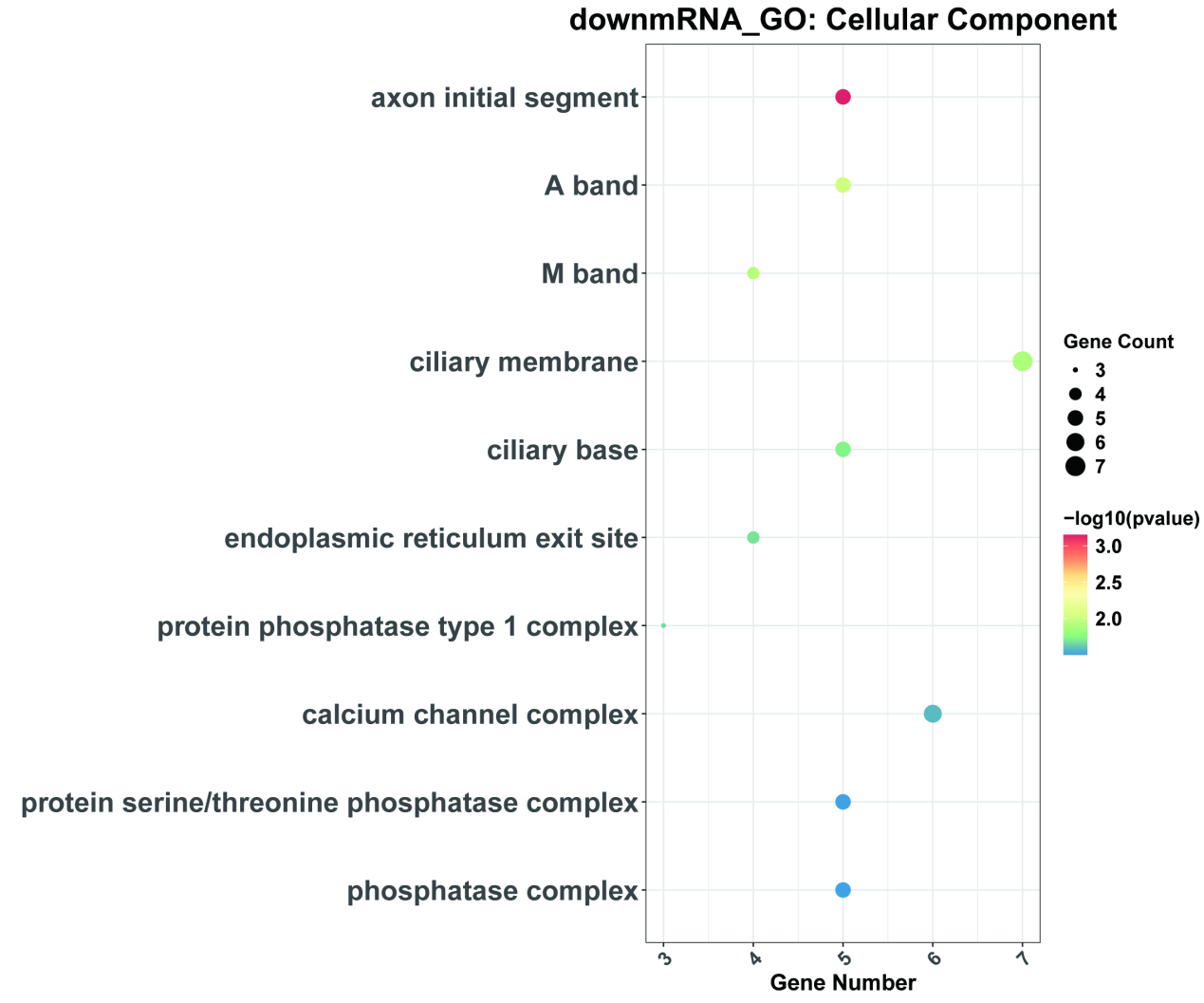
**Supplementary Figure S8. GO Cellular Component (CC) enrichment analyses for the downregulated mRNAs.**


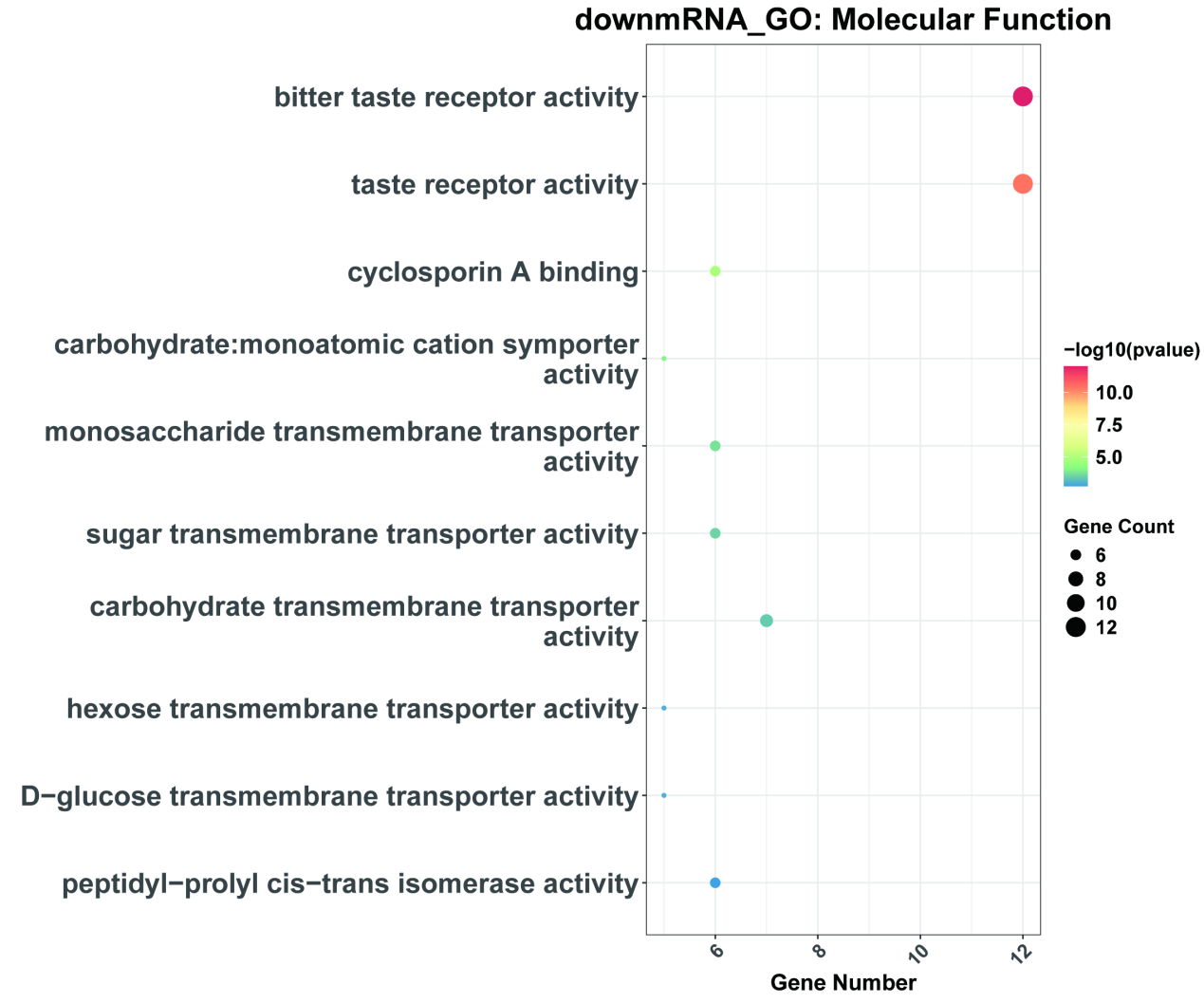


**Supplementary Figure S9. GO Molecular Function (MF) enrichment analyses for the downregulated mRNAs.**


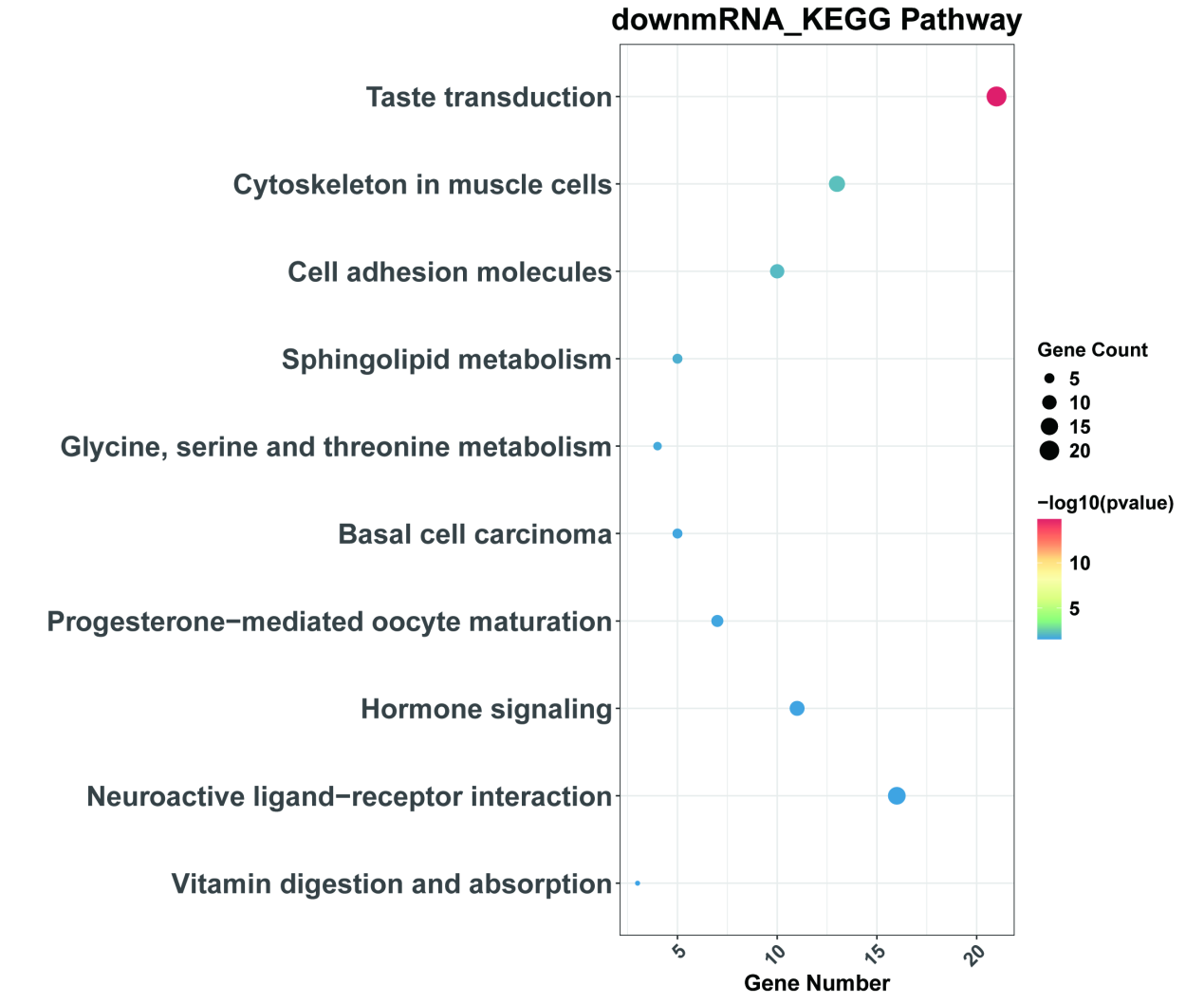


**Supplementary Figure S10. KEGG pathway enrichment analyses for the downregulated mRNAs.**


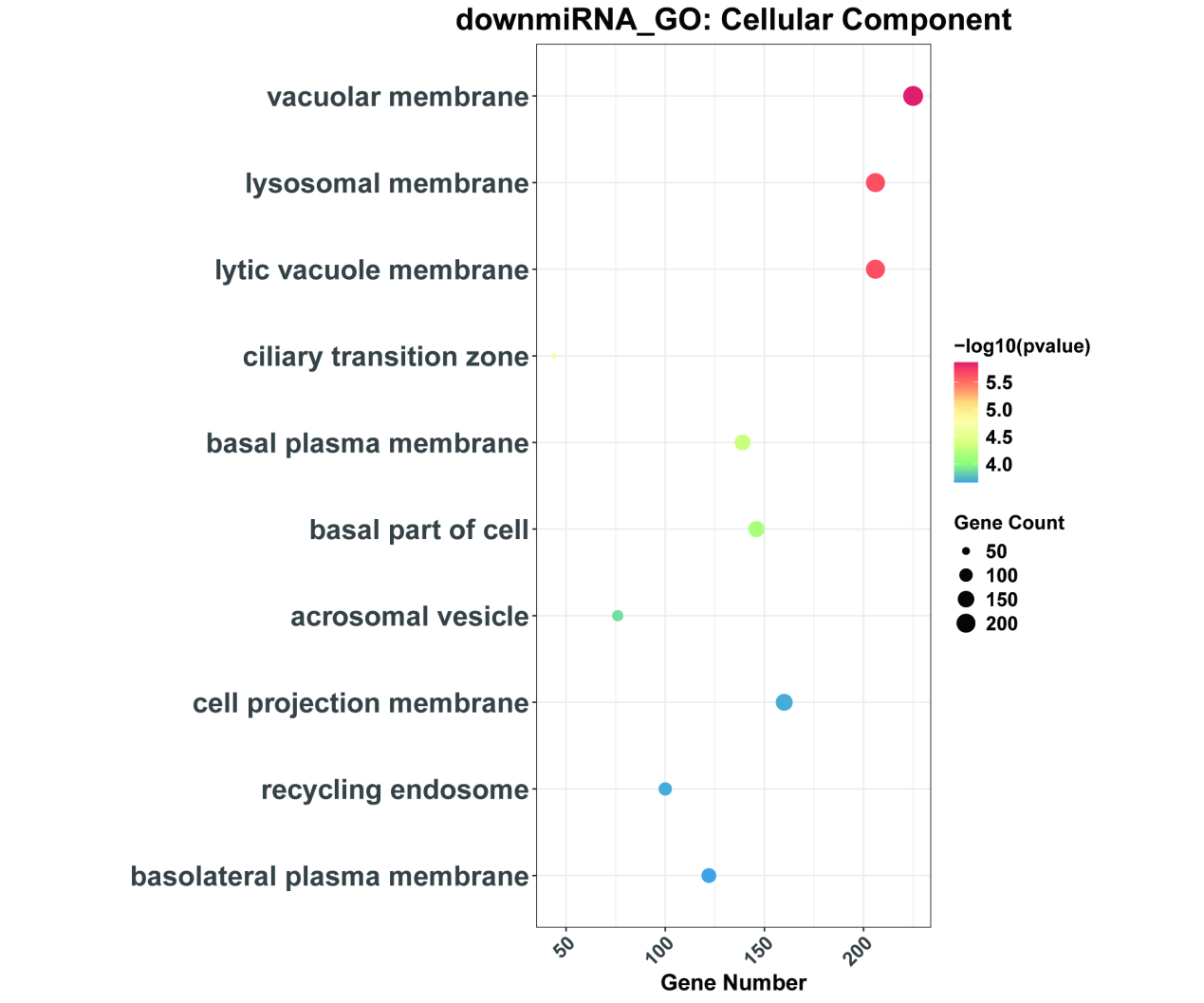


**Supplementary Figure S11. GO Cellular Component (CC) enrichment analyses for the downregulated miRNAs.**


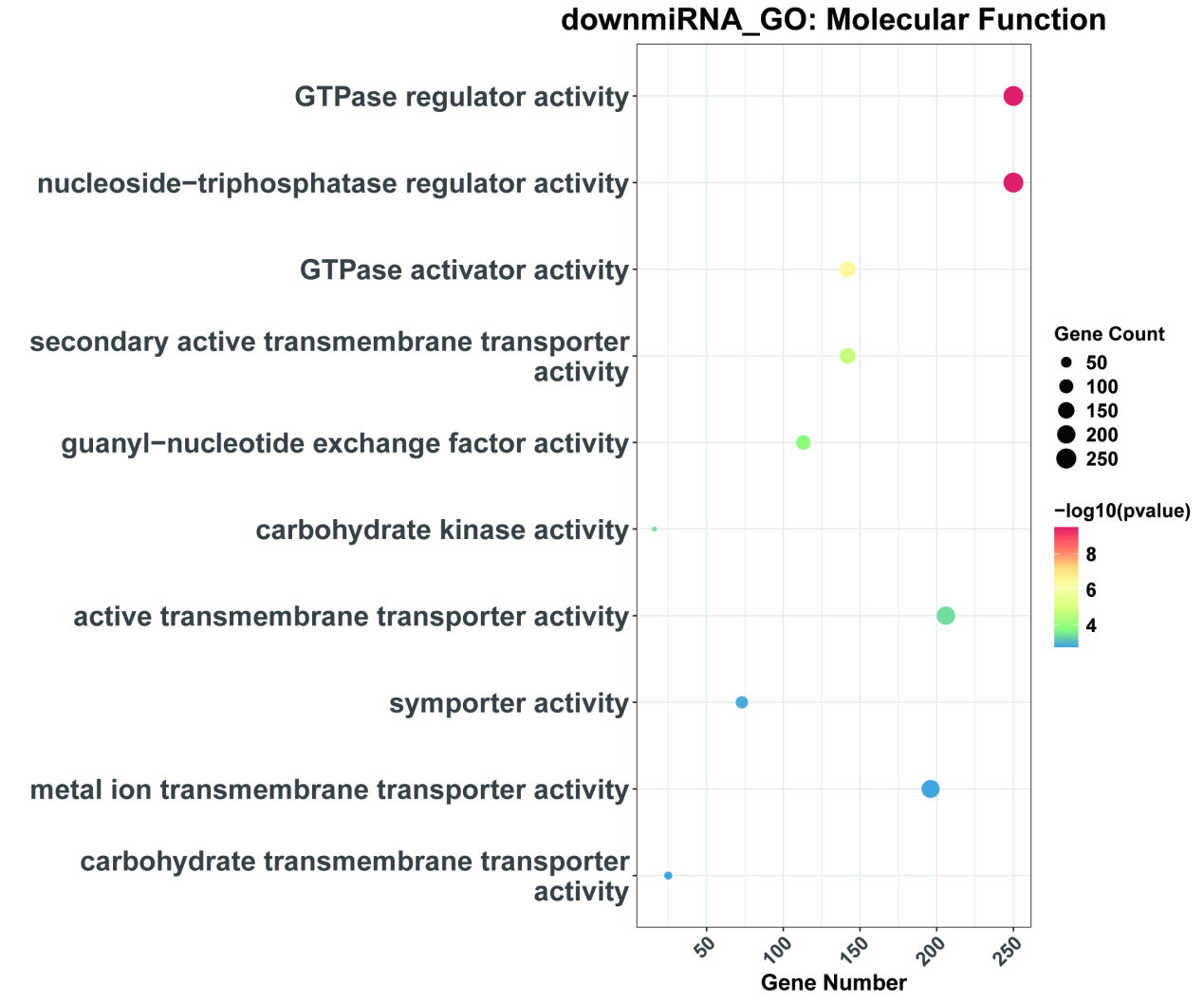


**Supplementary Figure S12. GO Molecular Function (MF) enrichment analyses for the downregulated miRNAs.**


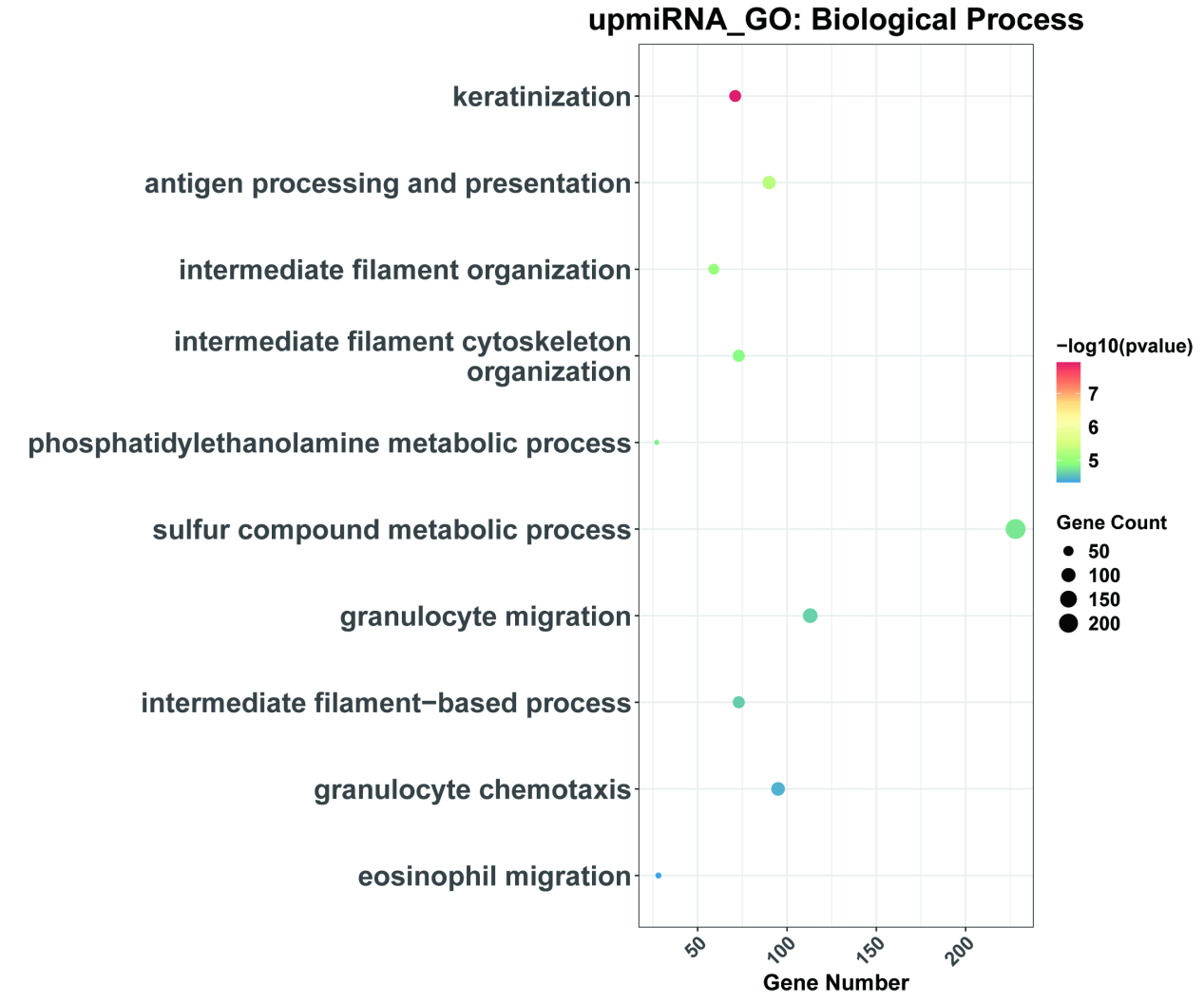


**Supplementary Figure S13. GO Biological Process (BP) enrichment analyses for the upregulated miRNAs.**


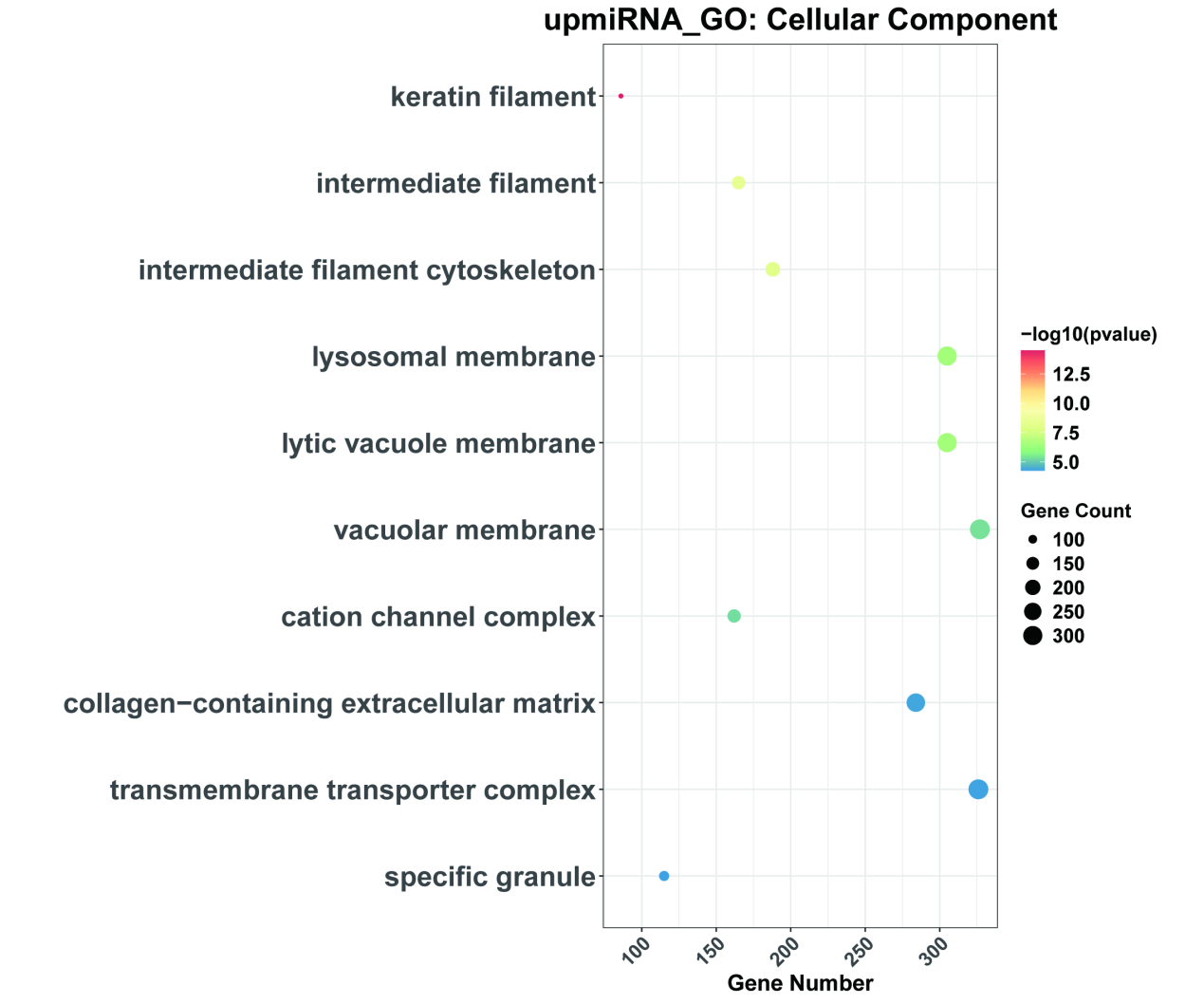


**Supplementary Figure S14. GO Cellular Component (CC) enrichment analyses for the upregulated miRNAs.**


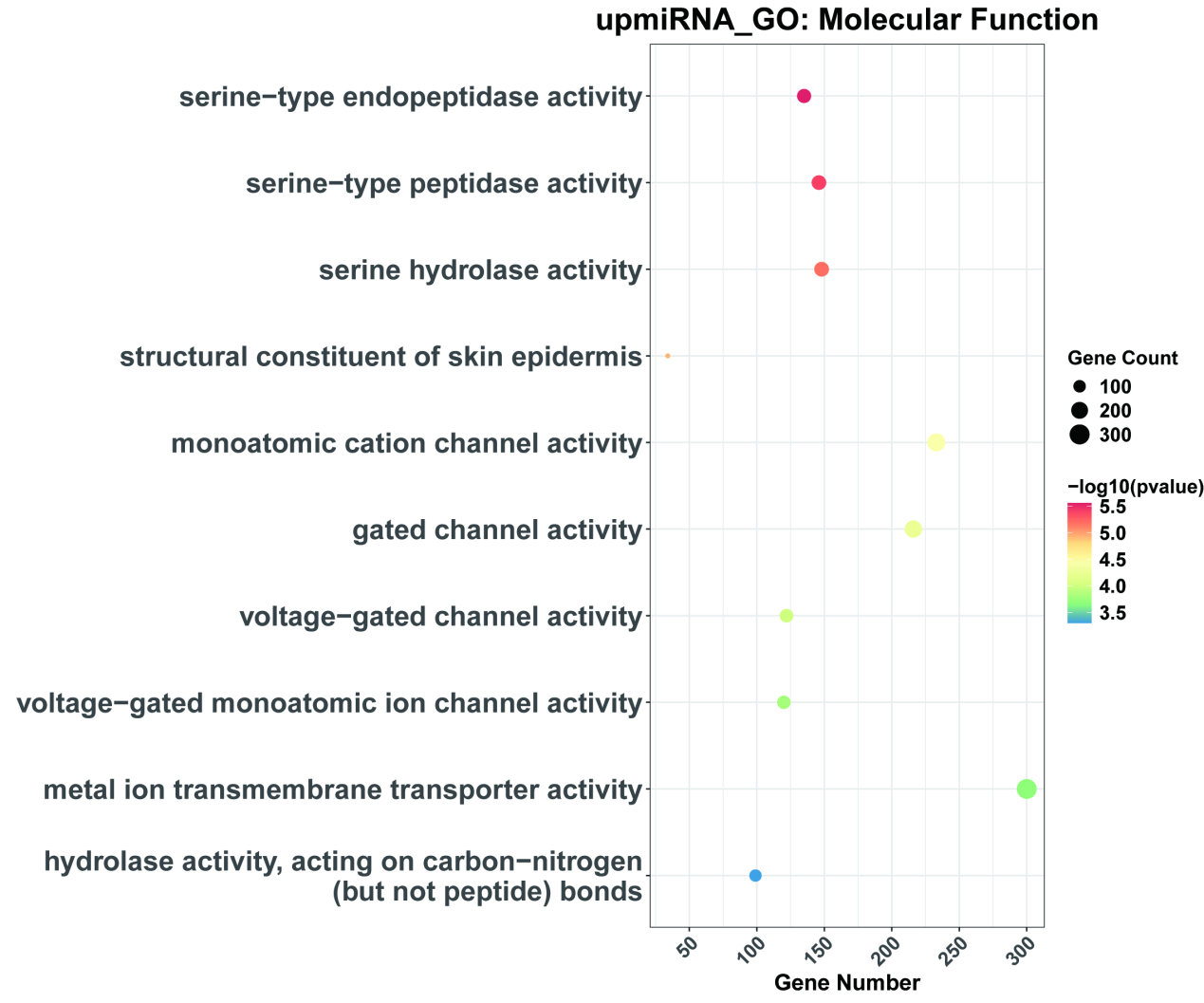


**Supplementary Figure S15. GO Molecular Function (MF) enrichment analyses for the upregulated miRNAs.**


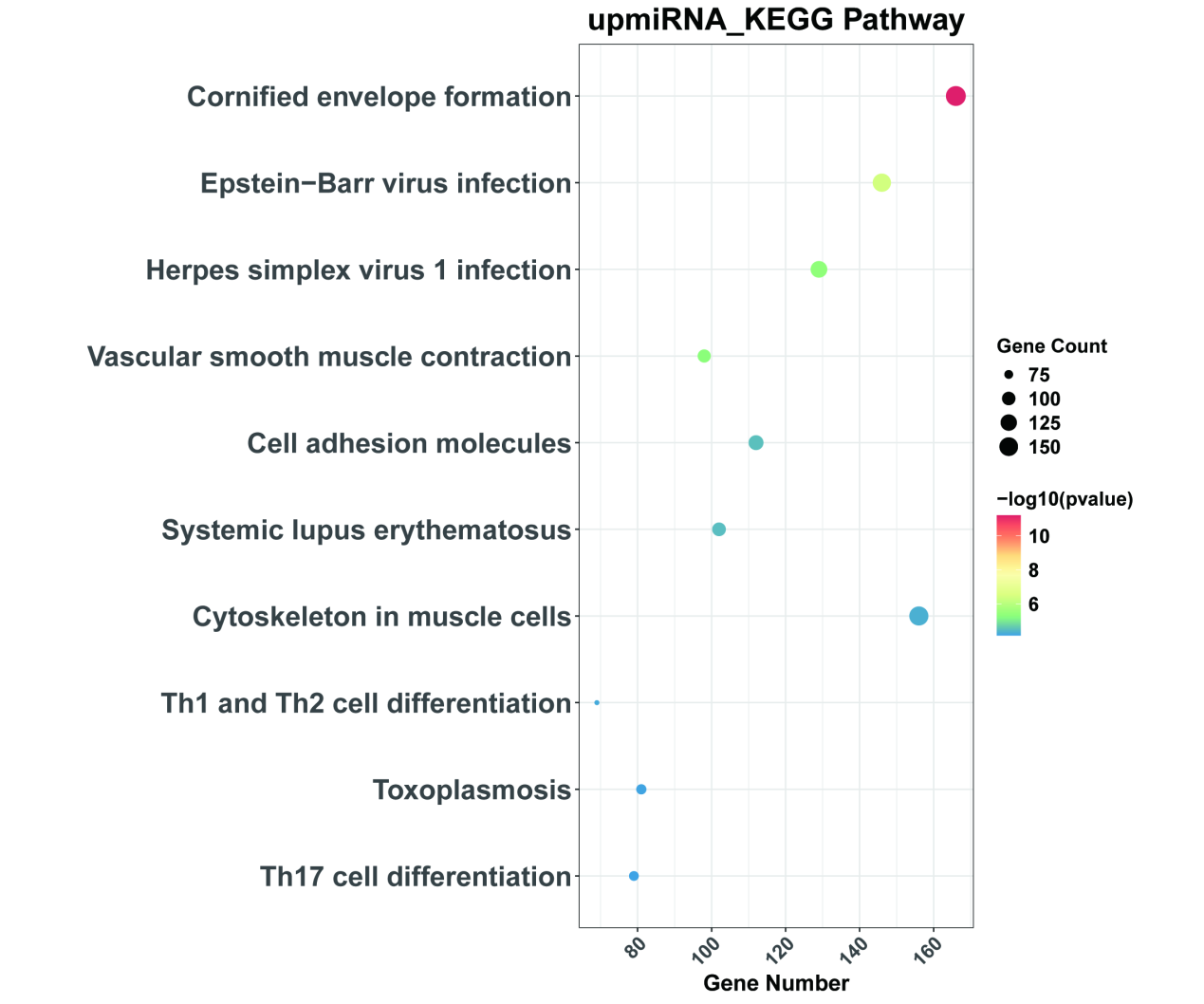


**Supplementary Figure S16. KEGG pathway enrichment analyses for the upregulated miRNAs.**


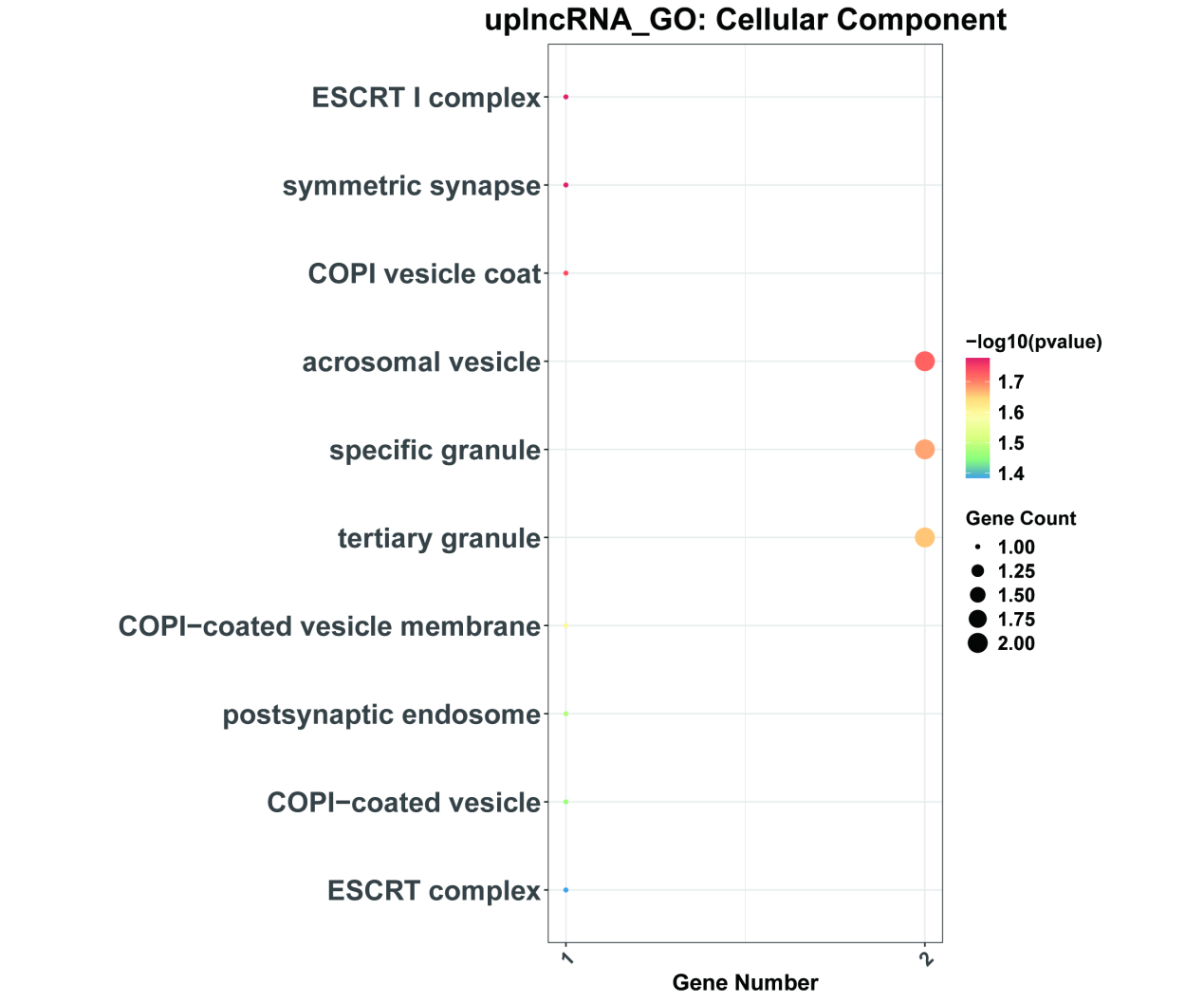


**Supplementary Figure S17. GO Cellular Component (CC) enrichment analyses for the upregulated lncRNAs.**


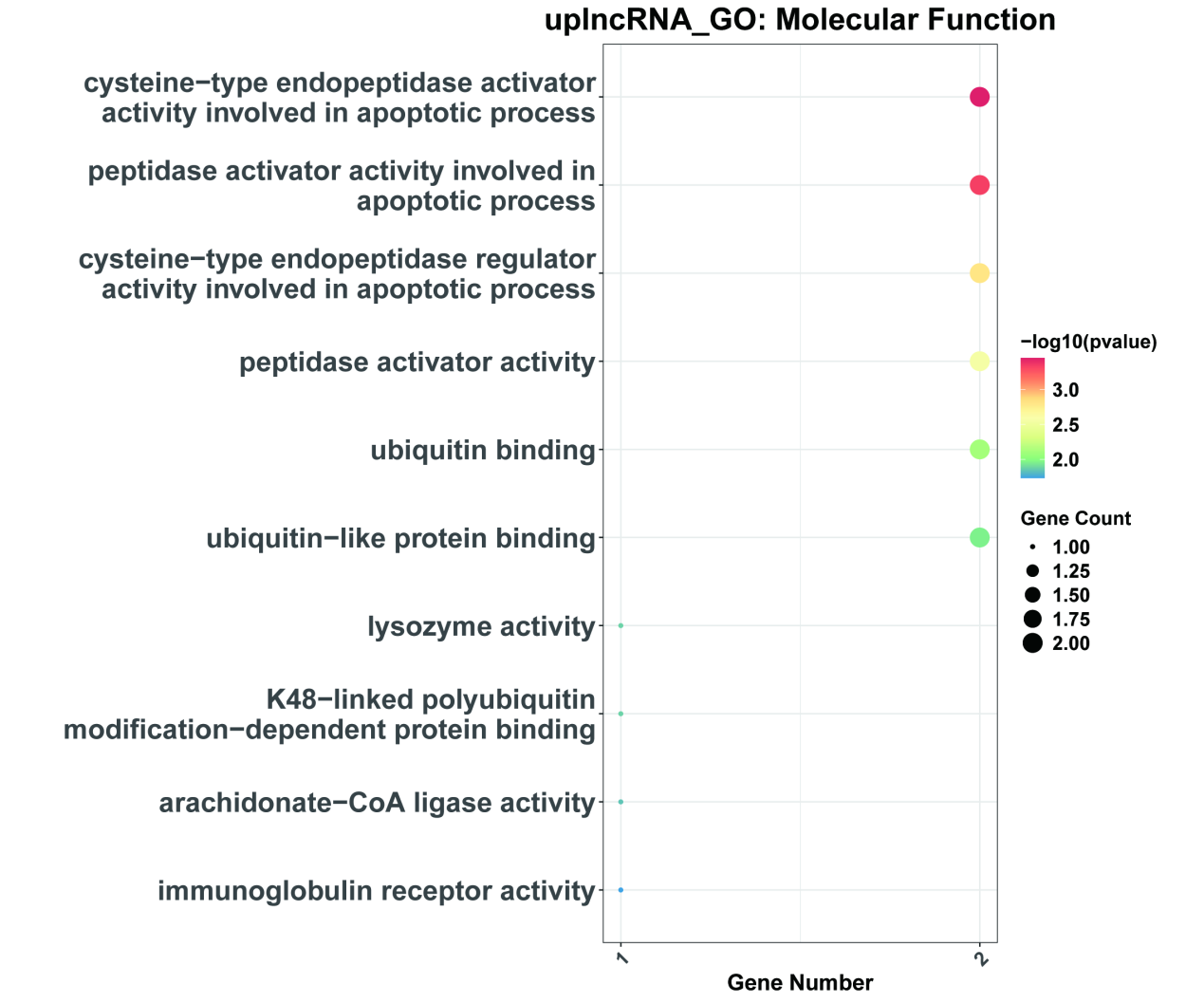


**Supplementary Figure S18. GO Molecular Function (MF) enrichment analyses for the upregulated lncRNAs.**


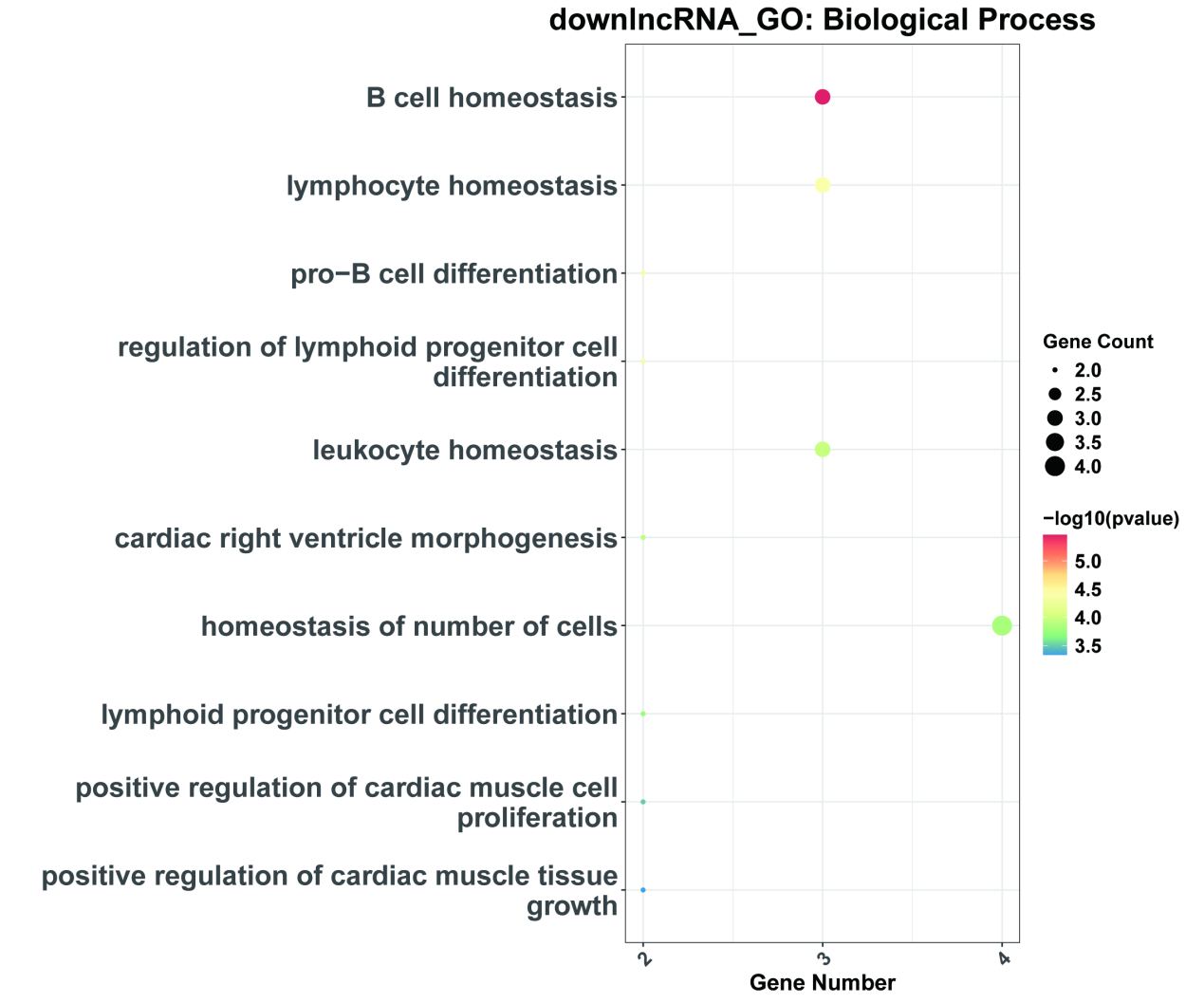


**Supplementary Figure S19. GO Biological Process (BP) enrichment analyses for the downregulated lncRNAs.**


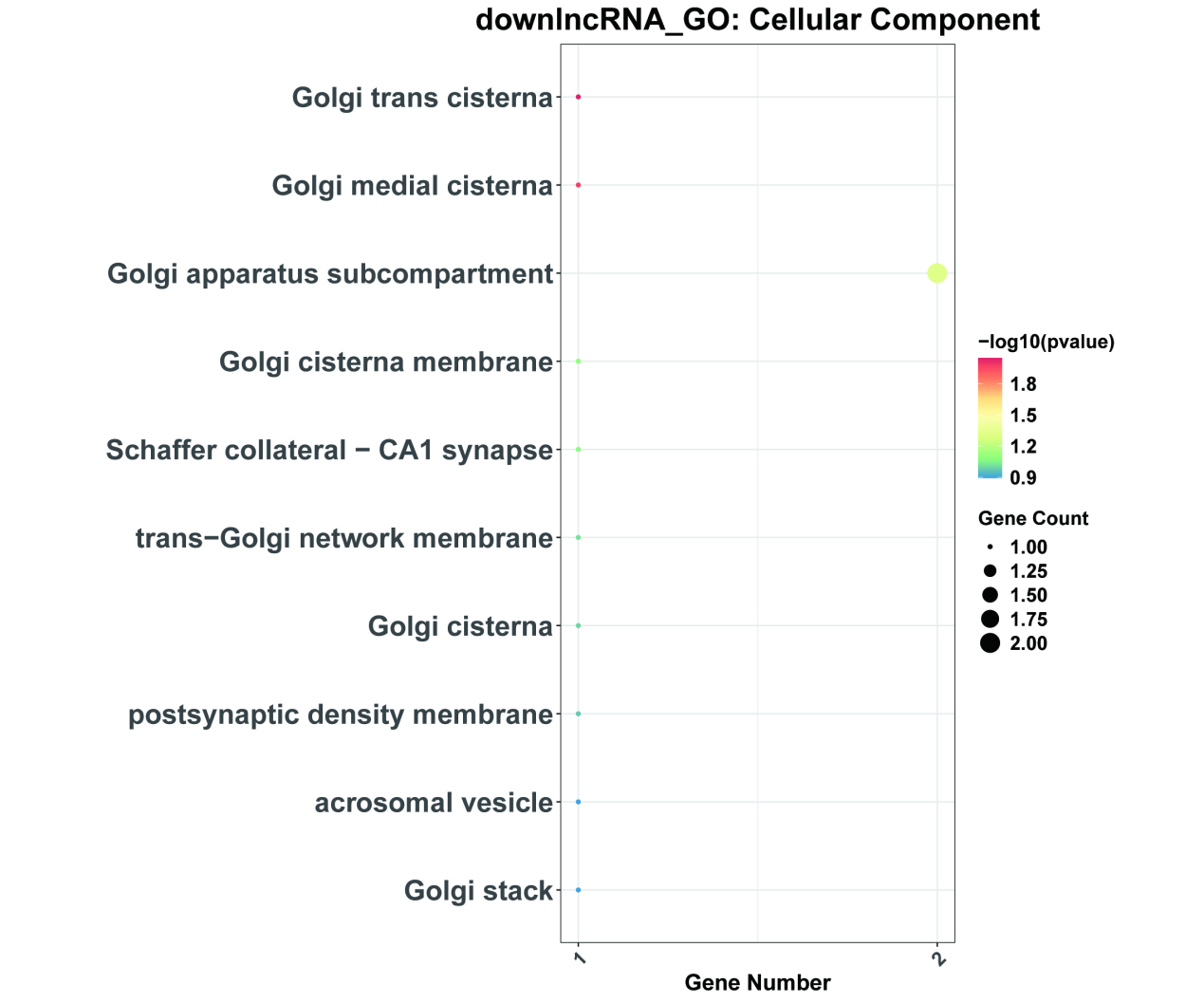


**Supplementary Figure S20. GO Cellular Component (CC) enrichment analyses for the downregulated lncRNAs.**


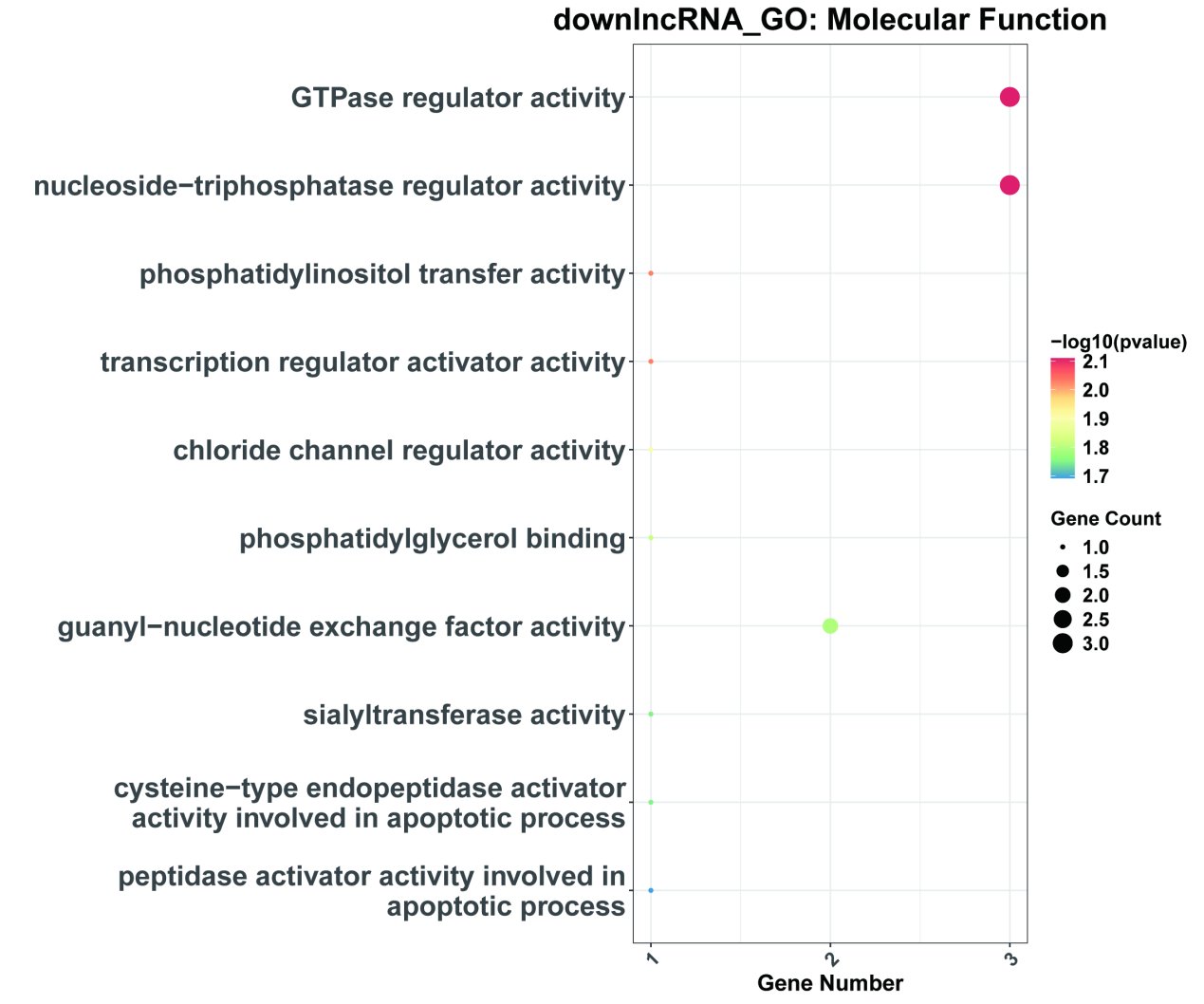


**Supplementary Figure S21. GO Molecular Function (MF) enrichment analyses for the downregulated lncRNAs.**


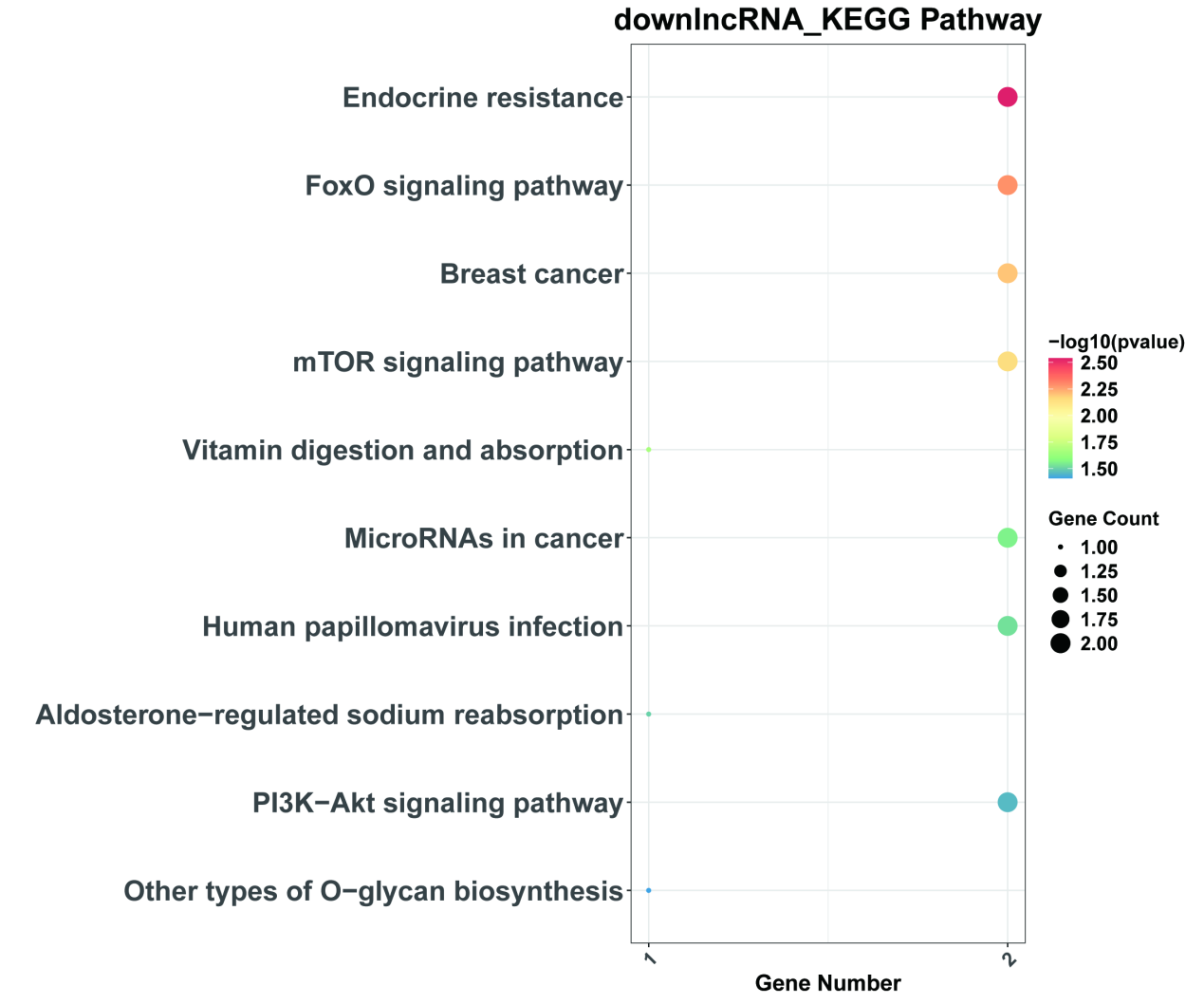


**Supplementary Figure S22. KEGG pathway enrichment analyses for the downregulated lncRNAs.**


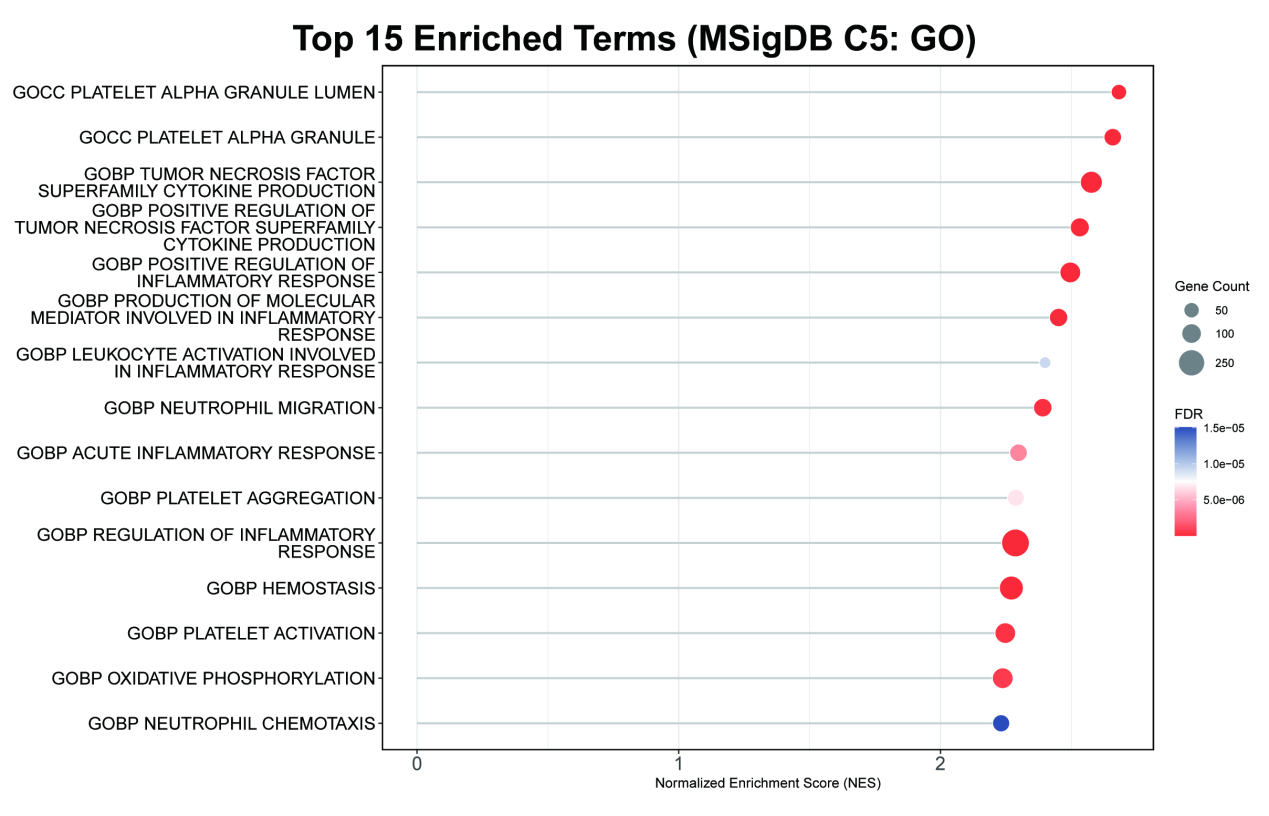


**Supplementary Figure S23. Visualization of the top 15 enriched Gene Ontology (GO) terms. The lollipop plot displays the top 15 significantly enriched pathways identified via GSEA using the MSigDB C5 (GO) collection. The x-axis represents the Normalized Enrichment Score (NES), and the y-axis lists the specific GO terms (including Biological Processes and Cellular Components). The size of each dot is proportional to the number of enriched genes (Gene Count), and the color gradient indicates the statistical significance (FDR; red represents higher significance).**


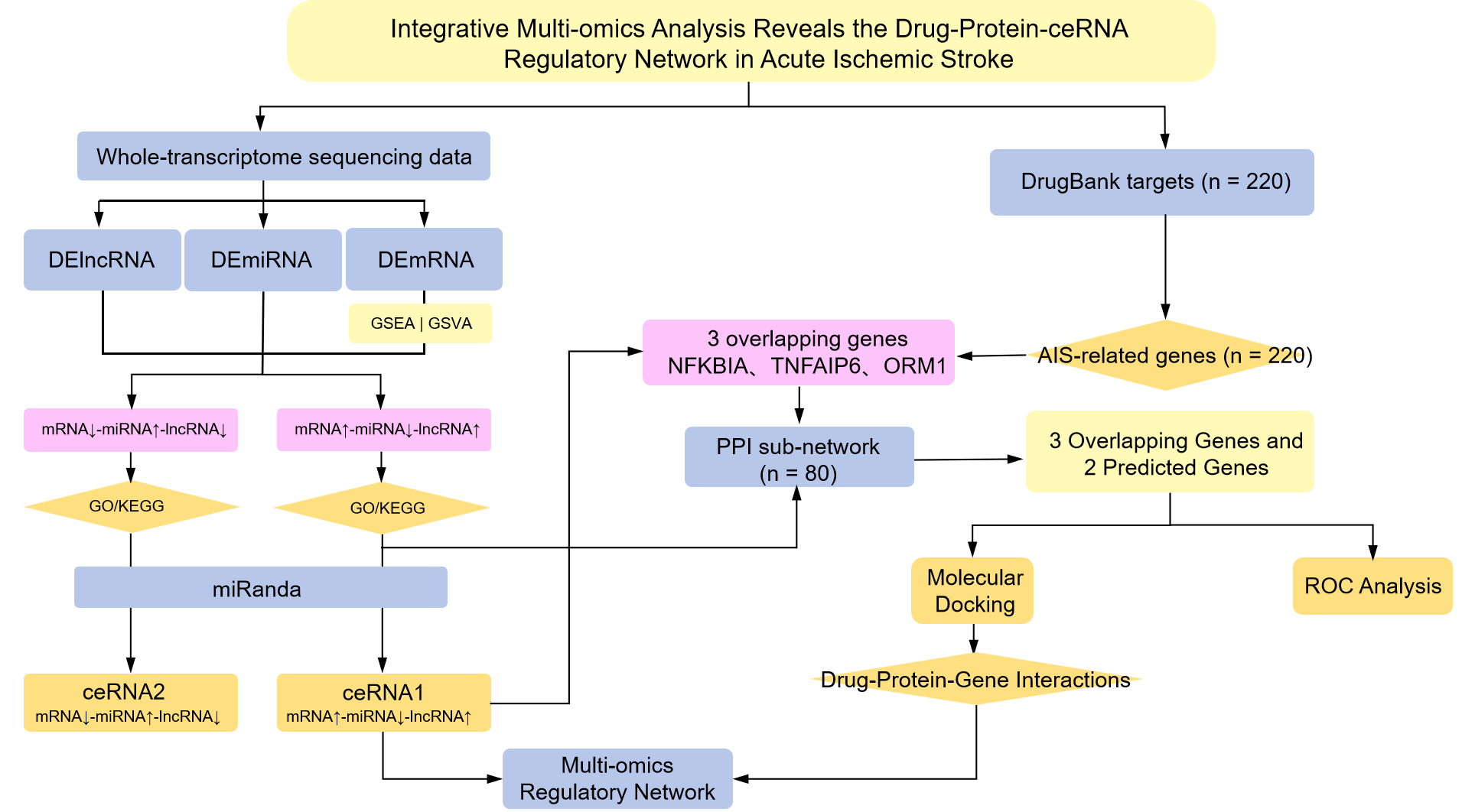


**Supplementary Figure S24. The workflow of this study.**
